# Imitating evolution’s tinkering by protein engineering reveals extension of human galectin-7 activity

**DOI:** 10.1007/s00418-021-02004-w

**Published:** 2021-06-21

**Authors:** Anna-Kristin Ludwig, Malwina Michalak, Adele Gabba, Tanja J. Kutzner, Donella M. Beckwith, Forrest G. FitzGerald, Gabriel García Caballero, Joachim C. Manning, Mark Kriegsmann, Herbert Kaltner, Paul V. Murphy, Maré Cudic, Jürgen Kopitz, Hans-Joachim Gabius

**Affiliations:** 1 Institute of Physiological Chemistry, Faculty of Veterinary Medicine, Ludwig-Maximilians-University Munich, 80539 Munich, Germany; 2 Institute of Pathology, Department of Applied Tumor Pathology, Faculty of Medicine, Ruprecht-Karls-University Heidelberg, 69120 Heidelberg, Germany; 3 School of Chemistry, National University of Ireland, Galway, H91 TK33 Ireland; 4 Department of Chemistry and Biochemistry, Florida Atlantic University, Boca Raton, FL 33431 USA; 5 Institute of Pathology, Department of General Pathology and Pathological Anatomy, Faculty of Medicine, Ruprecht-Karls-University Heidelberg, 69120 Heidelberg, Germany

**Keywords:** Calorimetry, Glycosylation, Lectin, p53, Proliferation, Protein design

## Abstract

Wild-type lectins have distinct types of modular design. As a step to explain the physiological importance of their special status, hypothesis-driven protein engineering is used to generate variants. Concerning adhesion/growth-regulatory galectins, non-covalently associated homodimers are commonly encountered in vertebrates. The homodimeric galectin-7 (Gal-7) is a multifunctional context-dependent modulator. Since the possibility of conversion from the homodimer to hybrids with other galectin domains, i.e. from Gal-1 and Gal-3, has recently been discovered, we designed Gal-7-based constructs, i.e. stable (covalently linked) homo- and heterodimers. They were produced and purified by affinity chromatography, and the sugar-binding activity of each lectin unit proven by calorimetry. Inspection of profiles of binding of labeled galectins to an array-like platform with various cell types, i.e. sections of murine epididymis and jejunum, and impact on neuroblastoma cell proliferation revealed no major difference between natural and artificial (stable) homodimers. When analyzing heterodimers, acquisition of altered properties was seen. Remarkably, binding properties and activity as effector can depend on the order of arrangement of lectin domains (from N- to C-termini) and on the linker length. After dissociation of the homodimer, the Gal-7 domain can build new functionally active hybrids with other partners. This study provides a clear direction for research on defining the full range of Gal-7 functionality and offers the perspective of testing applications for engineered heterodimers.

**Supplementary Information:**

The online version contains supplementary material available at 10.1007/s00418-021-02004-w.

## Introduction

Oligo- and polymers are the biochemical equivalent of (code) words written using the set of symbols (letters) of an alphabet. What has become basic knowledge for two alphabets (nucleotides and amino acids), i.e. their fundamental role in the flow of biological information, is gaining ground and momentum for carbohydrates. Explicitly, they are now considered to be letters of the sugar alphabet to “write” bioactive signals (glycans). Given their ubiquitous presence and structural diversity by well-elaborated biosynthesis, glycans are able to serve as a highly versatile pool of molecular messages (Sharon 1975; Buddecke 2009; Cummings 2009; Zuber and Roth 2009; Corfield 2017; Kopitz 2017). Experimentally, the histochemical glycophenotyping of cells and tissues by sugar receptors (lectins) from plants not only underscores the dynamic spatiotemporal regulation of cellular glycomes (Avrameas 1970; Spicer and Schulte 1992; Roth 1996, 2011; Manning et al. 2017a), but its successful application, e.g. by detection of histo-blood group ABH epitopes (Kilpatrick and Green 1992), conceptually provides an instructive model for specific glycan-lectin recognition in situ. Combined with the evidence for biological activity of plant lectins, e.g. as mitogens (Nicolson 1974; Borrebaeck and Carlsson 1989; Kaltner et al. 2018), the experience with phytohemagglutinin contributed to direct interest in investigating endogenous (tissue) lectins and to shaping the hypothesis of the sugar code, e.g. their involvement in the way an assumed “cell-surface code” may operate (Brandley and Schnaar 1986). In brief, lectins are assumed to read glycan-encoded information and translate it into cellular activity (Gabius and Roth 2017; Kaltner et al. 2019).

The detection and structural characterization of endogenous lectins revealed a large panel of such proteins. More than a dozen protein folds with the capacity for carbohydrate binding were identified, and this parameter of the resulting domains has become the distinctive characteristic for lectin classification (Gabius 1997; Kilpatrick 2002; Fujimoto et al. 2014; Solís et al. 2015). Divergence of the sequence of the carbohydrate recognition domain (CRD) after gene duplication led to the members of a lectin family. Of note, they can also differ in their quaternary structure and their modular architecture. Systematic structural analyses revealed that not all from the many theoretically possible modular arrangements are realized. This intriguing insight poses the pertinent riddle on why certain types of design, the result of evolution’s “molecular tinkering” (Jacob 1977), were favored during phylogenesis. Creating variants with the same CRD in different types of architecture for their comparative testing together with the wild-type protein is a means to address this challenge.

**Fig. 1:**
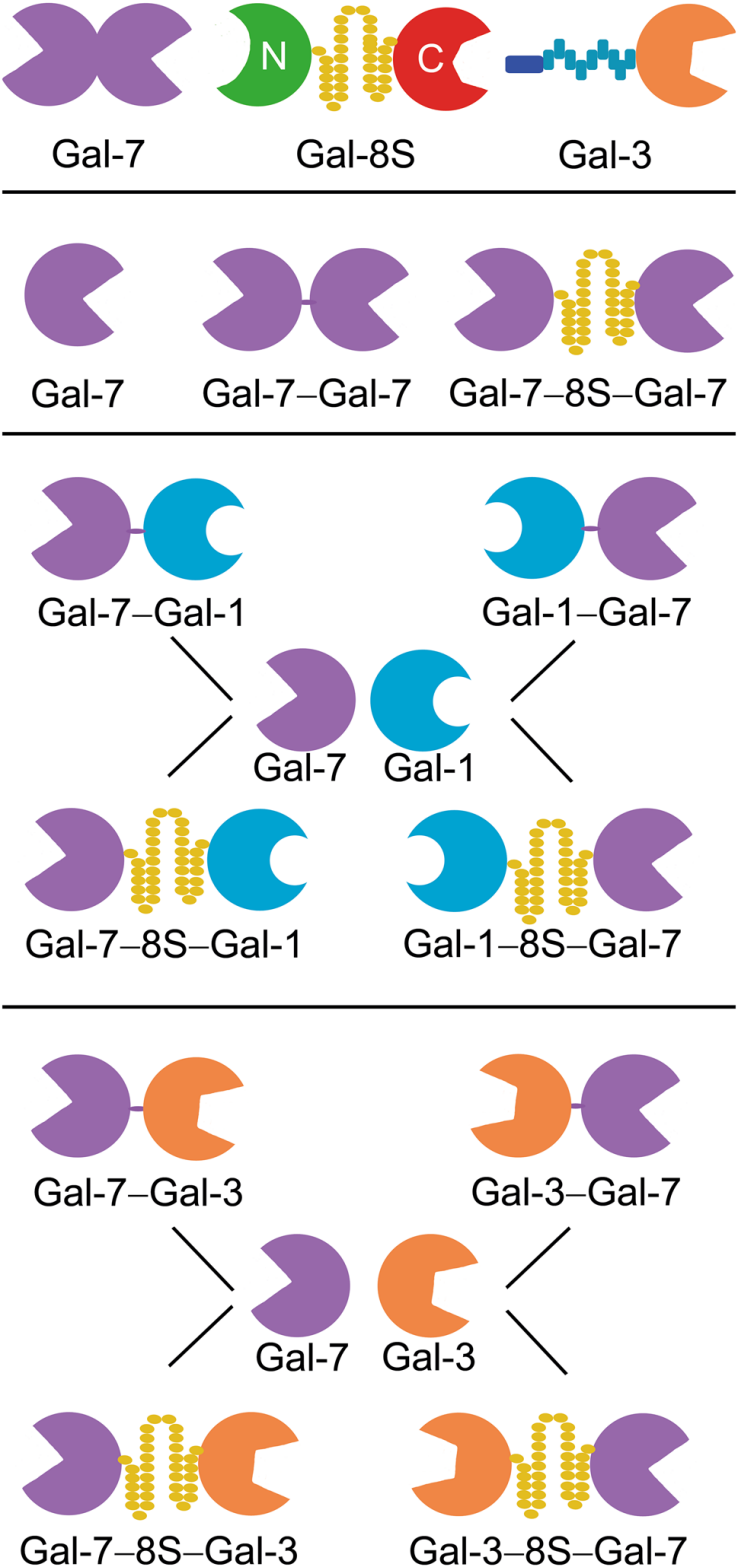
Modular architecture of the tested galectins: examples of the three types of vertebrate galectins (top row), the two homodimeric variants of Gal-7 established by direct or linker-mediated connection (middle) and the panel of heterodimers with directly conjugated CRDs and CRDs connected by the 33-amino-acid linker of Gal-8S (bottom)

Looking at the family of adhesion/growth-regulatory vertebrate galectins (Hirabayashi 1997, 2018; Thiemann and Baum 2016; Kaltner et al. 2017; de Jong et al. 2020; García Caballero et al. 2020a), their canonical β-sandwich-type CRD with its signature sequence for glycan contact is displayed in three types of structural arrangement: a non-covalently associated homodimer, a heterodimer held together by a linker, and the combination of a CRD with a structurally different second part (Fig. 1, top). Bridging glycan counter-receptors between cells or cells and extracellular matrix and on the cell surface is an efficient mechanism for selective contact-building and outside-in signaling. When protein engineering is applied to turn a wild-type (WT) galectin into variants within a clear hypothesis-driven frame of study, biochemical, histochemical and cell biological analyses will obtain relevant information to eventually answer the given question. In this report, we focus on galectin-7 (Gal-7) and initiate respective work.

This galectin was discovered in searches for marker proteins of keratinocyte differentiation (Madsen et al. 1995; Magnaldo et al. 1995). Upon further study, it proved to be a region-independent indicator of epithelial stratification (Magnaldo et al. 1998; Timmons et al. 1999). First insights into its physiological pluripotency, a general characteristic of galectins, were obtained by the measurement of a strong upregulation (i) by p53 reconstitution in a colon carcinoma cell (DLD-1) line (leading to its designation as p53-induced gene 1) and (ii) in UVB-induced sunburn keratinocytes that is associated with onset of apoptosis (Polyak et al. 1997; Bernerd et al. 1999). However, the aspect of context dependence and the likely versatility of its bioactivity were soon delineated by reports that chemically induced mammary carcinogenesis and progression toward an aggressive phenotype of thymic lymphoma, breast cancer and hypopharyngeal cancer in situ were accompanied by Gal-7 upregulation (Lu et al. 1997; Perou et al. 2000; Moisan et al. 2003; Saussez et al. 2006; Demers et al. 2010). Both WT and mutant p53, the latter in cooperation with NF-κB, have been seen to regulate expression of Gal-7 (Campion et al. 2013). The profile of Gal-7 gene regulation is most probably more variegated in humans, due to the presence of two genes (and promoters), than in the mouse and rat with a single gene (Kaltner et al. 2013).

In its role as cellular effector, the homodimeric lectin binds β-galactosides (e. g. *N*-acetyllactosamine (Lac*N*Ac) at *N*-glycan branch ends and internally in Lac*N*Ac oligomers as well as ganglioside GM1 pentasaccharide) (Ahmad et al. 2002; Hirabayashi et al. 2002; Kopitz et al. 2003; Dam et al. 2005; Iwaki and Hirabayashi 2018; Ludwig et al. 2019a). Moreover, it interacts with proteins, i.e. Bcl-2, E-cadherin and c-jun terminal kinase (JNK) (Villeneuve et al. 2011; Chen et al. 2016; Advedissian et al. 2017), and affects gene expression signatures (Kuwabara et al. 2002; Demers et al. 2005; Saussez et al. 2009; Bibens-Laulan and St-Pierre 2017). Dissociation of the homodimer accounts for the presence of free CRD that has a second fate beyond reassociation. The recently reported evidence for hybrid formation between the CRD of Gal-7 and the CRD of either Gal-1 or Gal-3 by nuclear magnetic resonance (NMR) spectroscopic and cell biological experiments (Miller et al. 2018) makes cooperation of these CRDs by a molecular association possible. The natural design of Gal-7 (with its two non-covalently associated CRDs) appears to allow such CRD switching and may thereby be physiologically preferable to a stable (covalent) CRD conjugation to build the homodimer. Thus, the questions arise as to whether and how (i) introducing a linkage between the two CRDs of Gal-7 to turn the non-covalently into a covalently connected homodimer and (ii) designing Gal-7-based heterodimers with the CRD of either Gal-1 or Gal-3 will change properties relative to the WT protein(s).

To resolve these two issues, we engineered cDNAs to obtain galectin variants accordingly using direct CRD conjugation and insertion of the 33-amino-acid linker of Gal-8 as natural spacer between the CRDs, optimized recombinant protein expression, performed affinity chromatography and rigorously characterized the purified variant proteins biochemically. Binding properties were ascertained and systematically studied for the canonical ligand Lac*N*Ac by isothermal titration calorimetry (ITC). Interactions with cellular binding partners were examined by galectin histochemistry in tissue sections including the WT proteins as control. Cell assays were finally performed to determine an impact of variant design on the activity of these galectins as regulator of proliferation in vitro. In order to keep the number of variables small, neuroblastoma (SK-N-MC) cells were used, in which ganglioside GM1 is the common counter-receptor for Gal-1, Gal-3 and Gal-7 (Kopitz et al. 1998, 2001, 2003).

## Materials and methods

### Protein engineering and production

A three-step polymerase chain reaction (PCR) procedure developed for the design of cDNA coding for Gal-1 with its two CRDs connected by the 33-amino-acid linker of Gal-8S was adapted to create the respective products (Vértesy et al. 2015). Restriction sites for *Nde*I and *Hind*III were strategically used in primer sequences (for details on the complete set of primer pairs, please see Supplementary Material, Table S1). In-frame ligation into the pGEMEX-1 (Promega, Mannheim, Germany) and the pET24a expression vectors (Novagen, Munich, Germany) was followed by systematically confirming the planned sequence integrity by cDNA sequencing. Yields of protein production after transformation of *E. coli* BL21 or Rosetta (DE3)-pLysS cells (Promega) were optimized in each case by varying the conditions as compiled in Supplementary Material, Table S2, and proteins were purified to electrophoretic homogeneity by affinity chromatography on home-made lactosylated Sepharose 4B obtained after resin activation by divinyl sulfone as the main step, as given in detail elsewhere (Gabius 1990).

### Mass spectrometry measurements

Matrix-assisted laser desorption ionization (MALDI) time-of-flight (TOF) mass spectrometry (MS) was performed for measuring the molecular mass and for peptide fingerprinting of each protein, as previously described for variants of Gal-1 and Gal-3 (Kopitz et al. 2014; Vértesy et al. 2015). In brief, starting with a solution of galectin in water at a concentration of 100 pmol/µL and its dilution (1:5; v/v) with trifluoroacetic acid, mass determination was performed with double-layer sinapinic acid as matrix on a rapifleX Tissuetyper instrument (Bruker Daltonics, Bremen, Germany) in positive-ion linear mode. For peptide fingerprinting, 5 µg protein was digested with 50 ng trypsin and the resulting digest desalted using reversed-phase ZipTip C_18_ (Merck, Darmstadt, Germany), and samples were then spotted on a MALDI target plate covered by α-cyano-4-hydroxycinnamic acid as matrix. These measurements were carried out on Ultraflex I or rapifleX Tissuetyper instruments. Version 2.4 or 3.4 of flexControl (for instrument control) and flexAnalysis (for data processing) and version 3.0 or 3.2 of BioTools (for analyzing annotated spectra; all from Bruker Daltonics), respectively, were used. Settings for enzyme specificity included cleavage at the N-terminal of proline (Pro) and permitted missing up to two cleavage sites. Carbamidomethylation was a fixed parameter due to routine treatment of galectins with iodoacetamide to prevent loss of activity by oxidation of cysteine (Cys) residues.

### ITC measurements

Titrations were performed under standard conditions, as applied previously, to ensure comparability (Kutzner et al. 2019; Ludwig et al. 2019a). Removal of cognate sugar from protein preparations was guaranteed by dialysis over the course of 72 h with five buffer exchanges (2 L each). Briefly, 2 µL aliquots of ligand-containing solution (from a total of 36.4 µL) were injected per step into the galectin-containing solution in 20 mM phosphate buffer at pH 7.2 with 10 mM NaCl and 10 mM β-mercaptoethanol, starting at a volume of 200 µL. Injections were performed every 180 s at 25 °C and 500 rpm in a PEAQ-ITC calorimeter (Malvern, Westborough, MA, USA). Protein concentrations were based on absorbance applying a sequence-based extinction coefficient calculated with ExPASy ProtParam software. Data were routinely processed by MicroCal PEAQ-ITC analysis software with the one-set-of-sites/sequential models and a fitted offset parameter to account for potential background signal.

### Galectin histochemistry

About 5-µm-thick sections of fixed (Bouin’s solution) and paraffin-embedded specimens of epididymis and jejunum from four 6-week-old C57BL/6 mice were routinely processed, starting with rehydration and an incubation step with blocking solution [1% carbohydrate-free bovine serum albumin (BSA) in phosphate-buffered saline (20 mM, pH 7.2; PBS)] to saturate sites for nonspecific binding of protein, as described in detail elsewhere (Manning et al. 2017b, 2021; García Caballero et al. 2020b). Biotinylated galectins obtained by labeling with the *N*-hydroxysuccinimide ester derivative of biotin (Sigma-Aldrich, Munich, Germany) under activity-preserving conditions (Gabius et al. 1991) were applied first, and their binding was made visible with Vectastain^®^ ABC Kit and Vector^®^ Red reagents (Biozol, Eching, Germany). Systematic titrations for WT and variant galectins were carried out to map staining profiles at minimal background intensity (ranges of tested concentrations are listed in Supplementary Material, Table S3). Inhibition by cognate sugar was examined in each case by running titrations of increasing concentrations of lactose (Lac) in serial sections of both organs. In addition to the free sugar, two Lac-presenting glycoclusters, i.e. (1) a divalent stilbene-based carrier and (2) a tetravalent tetraphenylethylene scaffold (for structures, please see Supplementary Material, Fig. S1; for details on synthetic procedures, please see Kutzner et al. 2019), were tested. Photomicrographs (pixel dimensions: 1338 × 1040, image bit depth: 36) were recorded with an Axio Imager M1 microscope (objective EC Plan-Neofluar 40x/0.75 Ph 2 M27; Carl Zeiss Microscopy, Göttingen, Germany) equipped with an AxioCam MRc3 digital camera (Carl Zeiss Microscopy), and the data sets then processed using AxioVision software (version 4.9; Carl Zeiss Microscopy). Assessment of staining was done independently by two observers. Intensity of staining is grouped into the following categories: −, no staining; (+), very weak but significant; +, weak; ++, medium; +++, strong; ++++, very strong.

### Cell growth regulation

Human neuroblastoma cells (strain SK-NM-C) were grown in 96-well tissue plates (Greiner, Nürtingen, Germany) at 37 °C for 48 h in Eagle’s minimal essential medium with 10% fetal calf serum (Thermo Fisher Scientific, Dreieich, Germany) and non-essential amino acids in the absence (control) and in the presence of 125 µg/mL galectin (in four independent experimental series run in triplicate per protein from aliquots of the same cell suspension per assay series). The cell count was determined using the reagents of the CellTiter 96 kit (Promega), and the data obtained for the controls and the WT and variant proteins were statistically processed by *t* tests, as described previously (Kopitz et al. 2003, 2017; Ruiz et al. 2014). Competition assays with WT Gal-3 as inhibitor were performed by adding this protein at a 10:1 ratio to solutions containing test galectins, as performed previously (Kopitz et al. 2001, 2003).

## Results

### The panel of Gal-7-based variants

Gal-7 is a homodimer constituted by the non-covalent association of its CRDs (Fig. 1, first row, left). Molecular engineering built a stable bridge between the two CRDs so that a tandem-repeat architecture is generated either without adding new amino acids (Gal-7–Gal-7) or by inserting the 33-amino-acid linker of Gal-8S (Gal-7–8S–Gal-7) (Fig. 1, second row). Using the CRDs of Gal-7 and Gal-1 (or Gal-3) as molecular bricks for heterodimer design, corresponding conjugations were directly performed between the C- and N-terminal positions. Of course, it was taken into account that two sequential constellations of the CRDs from N- to C-termini are now possible, as illustrated in the bottom part of Fig. 1.

**Fig. 2:**
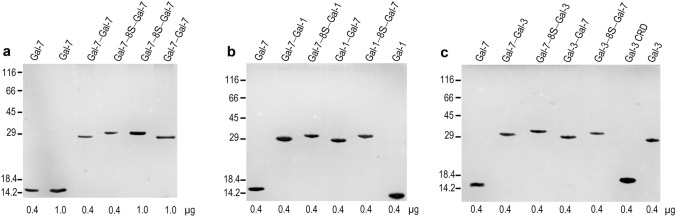
Illustration of gel electrophoretic analysis of Gal-7 (control) along with its homodimeric variants (**a**) or with its heterodimeric variants containing the CRD of Gal-1 (**b**) or of Gal-3 (**c**). Positions of marker proteins for mass calibration and quantity of protein are given

**Fig. 3:**
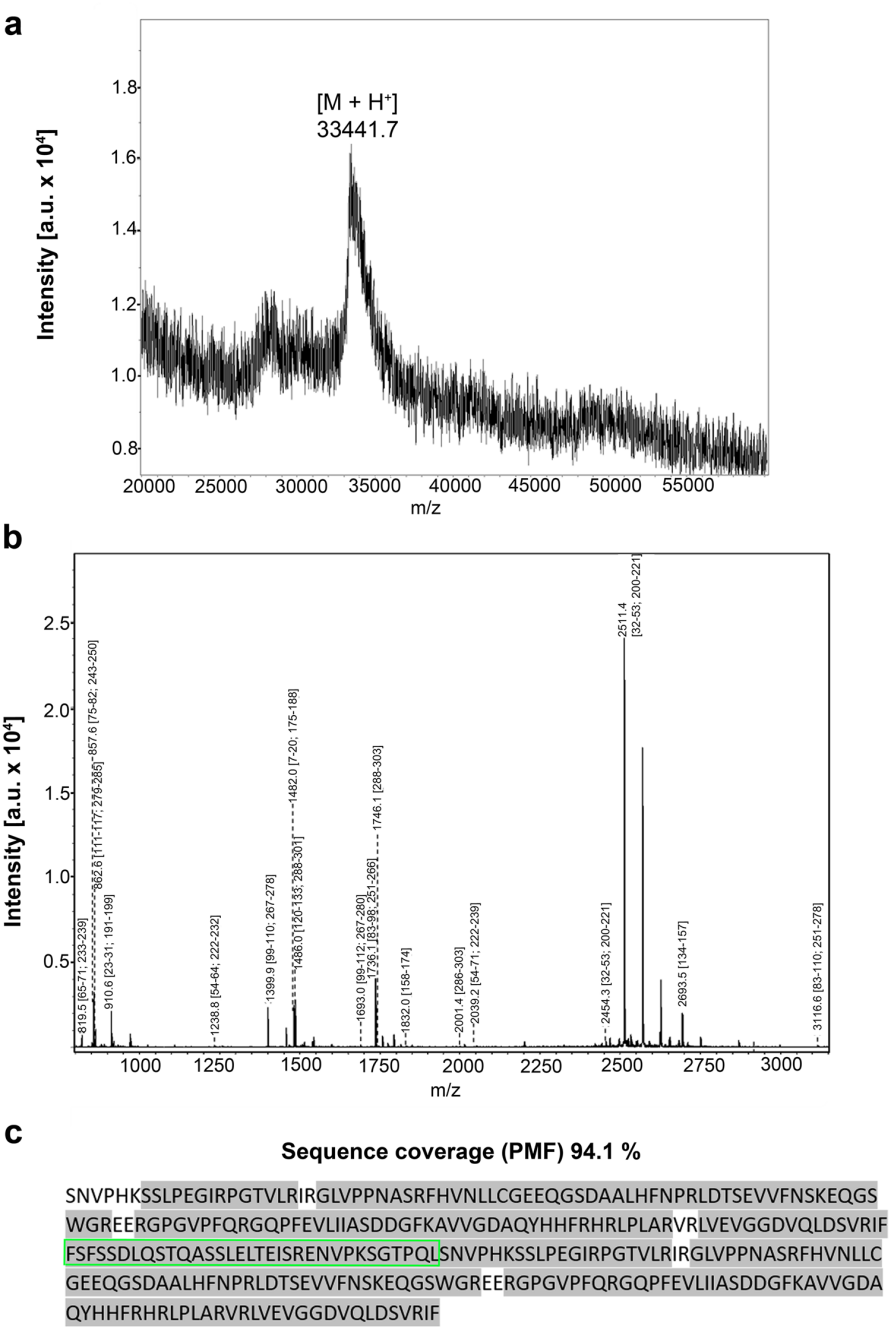
Mass spectrum of Gal-7–8S–Gal-7 (**a**), peptide mass fingerprint (**b**) and illustration of sequence coverage by fingerprinting that reaches a value of 94.1% (**c**). Sequence of the inserted 33-amino-acid linker of Gal-8S is highlighted in green

The insertion of the engineered cDNA sequence into expression vectors and their employment in recombinant production revealed template-driven biosynthesis of the variants in bacteria, these changes in Gal-7 design thus causing no obstacles to obtaining the new Gal-7 versions as proteins at high yield (details are given in Supplementary Material, Table S2). Chromatographic purification of the proteins on a Lac-bearing resin was possible, revealing proper function of the CRD, and led to pure proteins, first shown by documentation of respective data recorded after gel electrophoretic separation and protein staining (Fig. 2). The systematic MS analyses confirmed purity, determined the mass precisely and provided mapping on the level of tryptic peptides to reach high-level sequence coverage in all cases (for the example of the homodimer Gal-7–8S–Gal-7, please see spectra of mass determination (a) and the peptide fingerprint (b) together with the illustration of the sequence coverage (c) in Fig. 3 as well as the biochemical characteristics of the individual cleavage peptides in Table S4; for data sets of the characterization of all engineered variants, please see Supplementary Material, Figs. S2–S10 and Tables S5–S13). In order to ensure that both CRDs in the homo- and heterodimers are active, we performed ITC titrations of binding sites of the lectins up to their saturation by stepwise increases in the concentration of the canonical carbohydrate ligand Lac*N*Ac in the galectin-containing solution.

### Binding properties: ITC

**Fig. 4:**
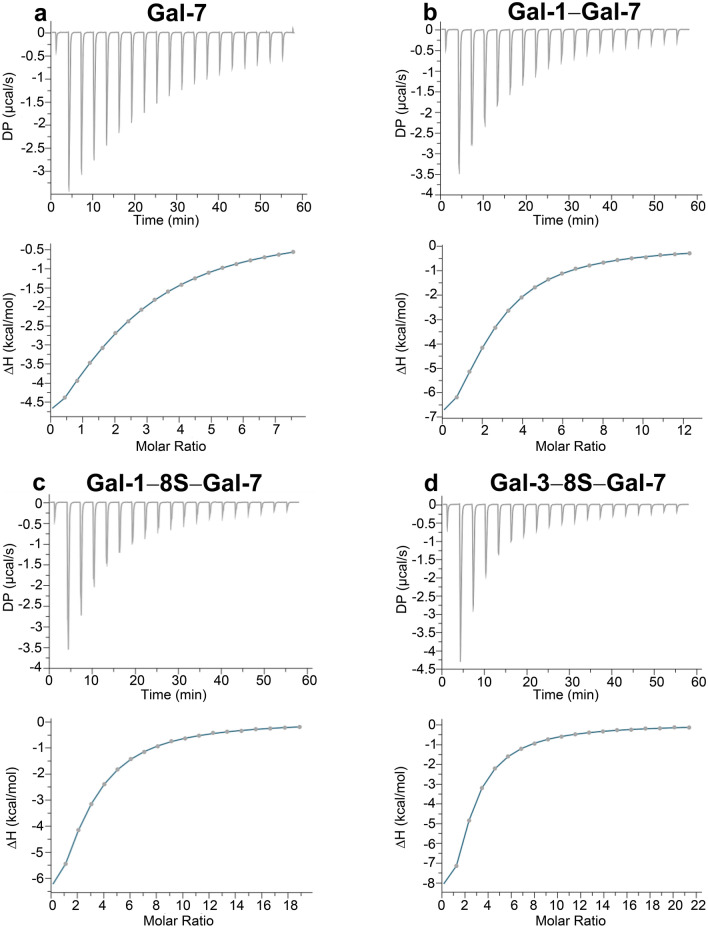
Thermograms of Lac*N*Ac (6 mM) association to human Gal-7 (**a**) and to the heterodimers Gal-1–Gal-7 (**b**), Gal-1–8S–Gal-7 (**c**) and Gal-3–8S–Gal-7 (**d**)

Since the solubility of the proteins and the presence of residual carbohydrate used to elute galectin from the ligand attached to the resin during affinity chromatography, i.e. Lac, are critical parameters for these experiments, we first worked with galectin preparations that were processed by lyophilization or precipitation with ammonium sulfate for storage and shipment and studied their behavior comparatively. In general, lyophilized material proved better suited for analysis, and no precipitation of protein occurred during the period of 72 h of dialysis to remove traces of Lac. Data of exemplary titrations, i.e. thermograms, are presented in Fig. 4 for the cases of WT Gal-7 and three variants.

These results were first processed by software based on the one-set-of-binding-site model. As presented in Table 1, each CRD of the homo- and heterodimers proved to be active, revealed by reaching a value of n of about 2 (binding sites per protein). Association of Lac*N*Ac was enthalpically driven, and this thermodynamic signature was maintained when the stoichiometry was deliberately set to 2 prior to processing data (Table 1). Performing respective experimental series and calculating thermodynamic parameters with the same software was extended from Lac*N*Ac to two further ligands. The data obtained with Lac (Table S14) corroborated the conclusions on activity. The core 1 mucin-type *O*-glycan disaccharide (Thomsen-Friedenreich (TF) antigen CD176) interacts with Gal-7’s CRD (Table S15, Fig. S11), as known for the Gal-3 CRD, much less so for Gal-1 (García Caballero et al. 2020b). These data indicate that CRDs that have different preferences among β-galactosides such as Gal-7 and Gal-1 can be brought together into a protein by the possibility of forming heterodimers.

**Table 1:** Summary of thermodynamics of association of Lac*N*Ac (6.0 mM) to human Gal-7 and its homo- and heterodimers with Gal-1 and Gal-3 at 25 °C calculated using the one-set-of-sites binding model

Lectin	[Cell] (μM)	*n*	*K*_a_ (× 10^4^ M^−1^)	−Δ*G* (kcal/mol)	−Δ*H* (kcal/mol)	−*T*Δ*S* (kcal/mol)	*K*_d_ (μM)
Gal-7	150	2.04	0.31	4.78	9.43 ± 0.140	4.66	316 ± 5.88
Gal-7–Gal-7^a^	60	2.05	0.38	4.89	1.99 ± 0.090	2.90	261 ± 6.21
	15	2.00 fixed	0.32	4.80	7.84 ± 0.088	3.04	305 ± 4.91
Gal-7–8S–Gal-7	38	1.92	0.35	4.83	6.01 ± 0.375	1.18	287 ± 9.55
Gal-7–Gal-1	32	2.00	0.97	5.44	10.70 ± 1.05	5.26	103 ± 6.11
	25	2.00 fixed	0.87	5.38	13.10 ± 0.108	7.73	114 ± 1.66
Gal-1–Gal-7	90	1.97	0.57	5.13	13.50 ± 0.145	8.37	175 ± 2.11
Gal-1–8S–Gal-7	60	2.02	0.61	5.17	14.80 ± 0.416	9.67	162 ± 3.79
Gal-1^c^	110	2.09	1.17	5.55	9.81 ± 0.08	4.26	85.2 ± 1.52
Gal-7–Gal-3	35	1.90	1.14	5.54	17.10 ± 1.63	11.6	86.5 ± 5.75
	50	2.00 fixed	1.72	5.78	10.30 ± 0.363	4.48	58.2 ± 5.22
Gal-7–8S–Gal-3^b^	20	1.88	3.03	6.13	9.15 ± 0.208	3.02	32.5 ± 0.81
Gal-7–8S–Gal-3	20	2.00 fixed	2.18	5.92	9.30 ± 0.164	3.38	45.9 ± 1.96
Gal-3–Gal-7	40	1.93	1.04	5.48	17.80 ± 0.509	12.3	96.2 ± 2.10
	50	2.00 fixed	1.40	5.66	12.10 ± 0.385	6.41	70.8 ± 5.29
Gal-3–8S–Gal-7	55	1.93	1.44	5.68	14.30 ± 1.05	8.59	68.6 ± 6.56
	55	2.00 fixed	1.26	5.60	14.40 ± 0.343	8.79	78.8 ± 4.29
Gal-3^d^	118	1.11	2.67	6.04	12.7 ± 0.07	6.65	37.5 ± 0.48
Gal-3 CRD^d^	90	0.97	2.19	5.92	9.70 ± 0.27	3.78	45.6 ± 1.64

^a/b^Concentration of Lac*N*Ac is 10 mM^a^ or 2 mM^b^, respectively

^c/d^From Kutzner et al. 2019^c^ and García Caballero et al. 2020b^d^

Since the levels of affinity to Lac*N*Ac/Lac and especially to the core 1 disaccharide differed among the three types of CRD, as shown for Lac*N*Ac in Table 1, it was reasonable to proceed to apply a model of sequential binding for calculating the thermodynamic parameters of heterodimers from the measured data. Indeed, this procedure resulted in obtaining two affinity constants with n values maintaining a value of about 2 (Supplementary Material, Table S16; respective data for the TF antigen are presented in Table S17). In principle, the association of a monovalent sugar such as Lac*N*Ac to the galectins thus engages both CRDs.

This set of measurements thereby fulfilled their purpose of revealing binding activity at both CRDs within this panel of bivalent proteins. As a consequence, systematic comparisons of the binding of the set of Gal-7 CRD-based proteins to cells could be performed. Considering tissue sections as an assay platform that presents different cell types and extracellular matrix for interaction, we next determined the staining profile of each protein shown to be functionally active at both CRDs under identical conditions on sections of fixed material from two types of organs.

### Binding properties: tissue sections

**Fig. 5:**
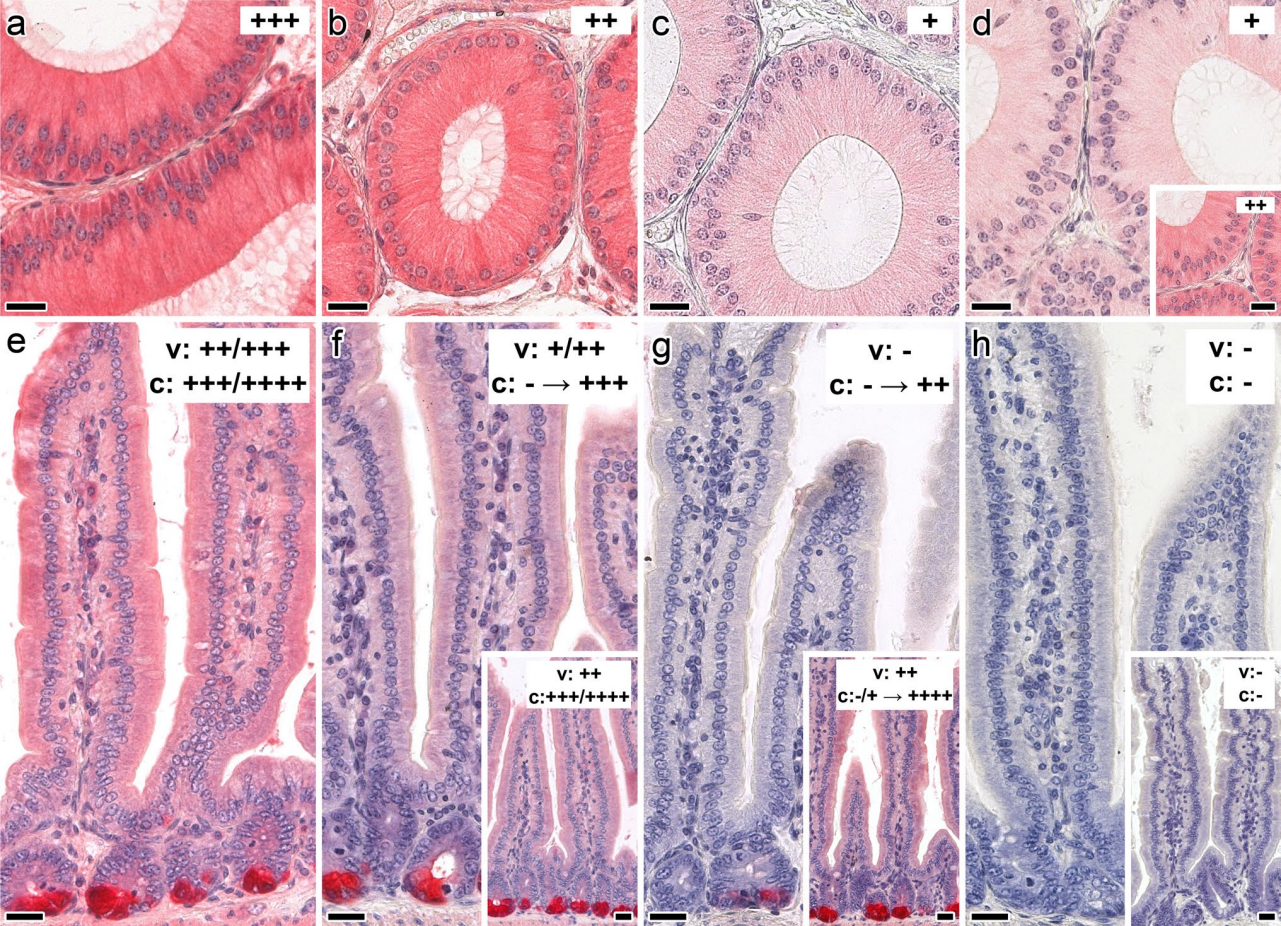
Illustration of the inhibitory effect of cognate sugar (Lac) on staining profiles with the biotinylated Gal-7–Gal-7 homodimer in cross sections through the fixed murine epididymis (initial segment; **a**–**d**) or jejunum (**e**–**h**). Strong staining intensity in the epididymis (**a**) was reduced in stepwise fashion by the presence of 0.2 mM or 5 mM Lac (**b**, **c**). Bivalent glycocluster (at 0.2 mM Lac) was more effective than tetravalent compound (**d**, inset to **d**). Galectin binding in the jejunum was blocked by Lac present in the bivalent glycocluster (control: **e**; sugar concentrations of 0.1 mM, 0.5 mM and 1 mM: **f**–**h**), insets to **f**–**h** showing respective activity of free Lac at 0.1 mM, 0.5 mM and 20 mM. The galectin was applied at 4 µg/mL. Scale bars are 20 µm

In the first stage of this part of the study, conditions that yield optimal signal-to-background ratio were identified in each case by systematic titrations. Having set this internal standard (for details, please see Supplementary Material, Table S3), the susceptibility of galectin binding to the presence of the canonical cognate sugar was evaluated. As illustrated exemplarily in Fig. 5 for sections of epididymis (panels a–d) and jejunum (panels e–h) and in Supplementary Material, Fig. S12, Lac was effective in reducing signal intensity, and its presentation by a bivalent scaffold markedly improved the inhibitory capacity at low concentrations. Testing galactose as very low-affinity binder and a panel of non-cognate mono- and disaccharides as osmolarity controls extended the evidence for inhibition of binding by specific glycan (Supplementary Material, Fig. S13).

**Fig. 6:**
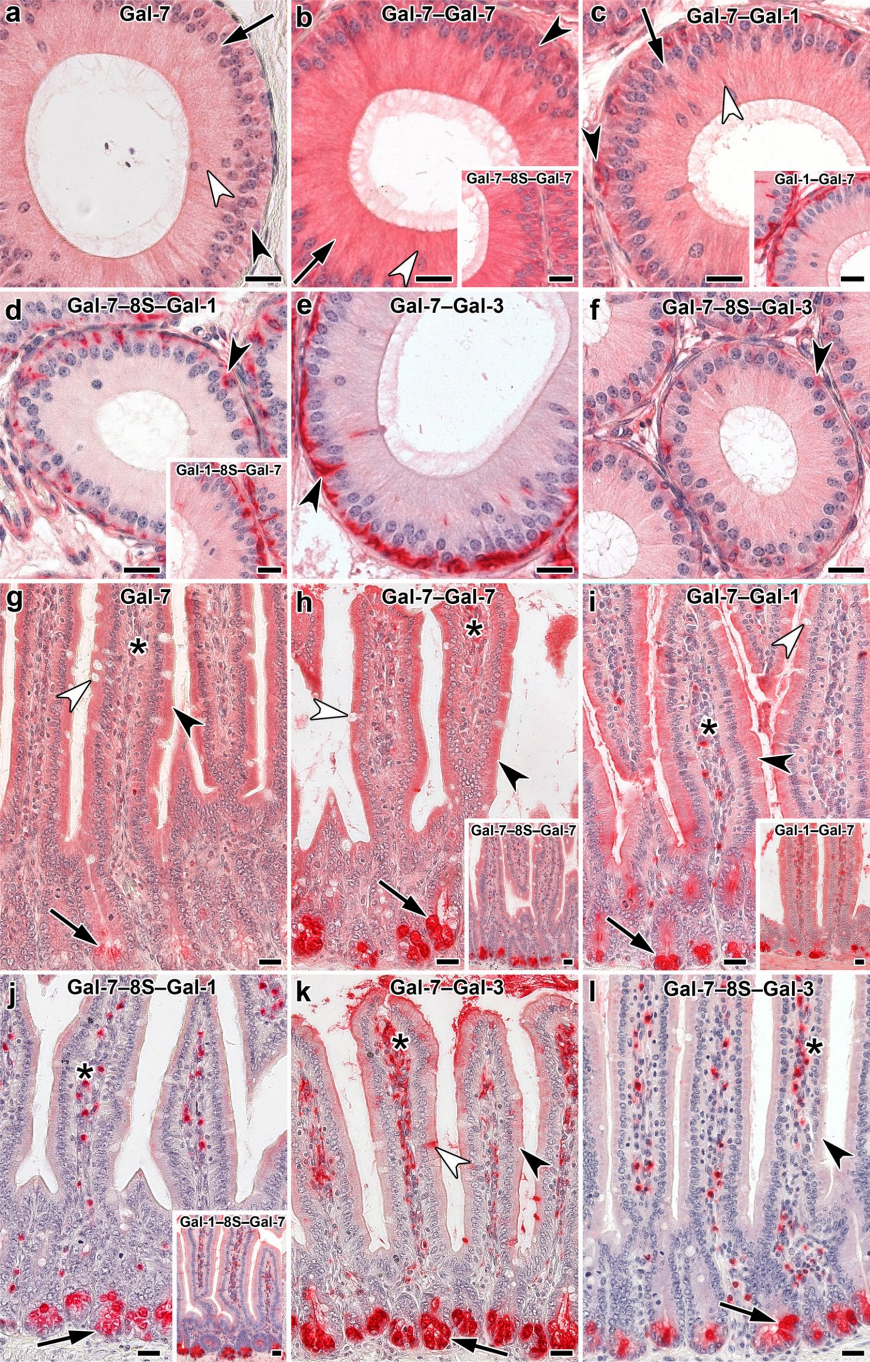
Illustration of staining profiles by labeled Gal-7 and Gal-7-based variants in cross sections of fixed murine epididymis (initial segment **a**–**f**) and jejunum (**g**–**l**). Extent of positivity appeared rather similar in principal (arrow), apical (white arrowhead) and basal (black arrowhead) cells with Gal-7 (**a**), its directly conjugated homodimer (**b**) and the Gal-7–Gal-1 heterodimer (**c**). Insertion of the peptide linker led to intensity reduction, strongly in this case and less so in the Gal-1–Gal-7 protein (**d** and inset; arrowhead: basal cell). The marked impact of linker presence on the signal intensity of basal cells (arrowhead) is documented in the case of the Gal-7–Gal-3 heterodimer pair (**e**, **f**). Murine jejunum presented similar staining profiles for Gal-7 (**g**), its directly linked homodimer (**h**) and the Gal-7–Gal-1 heterodimer (**i**); surface enterocytes (black arrowhead) and contents of goblet cells (white arrowhead), crypt cells (arrow) and lamina propria cells (asterisks) are highlighted. Presence of linker (inset to **h**) and change in CRD position in the dimer (inset to **i**) have minor effects. Presence of linker showed a strong reduction for the Gal-7–Gal-1 heterodimer (**j**), less so for its sequence permutation-based protein (inset). The same impact is observed for the Gal-7–Gal-3 heterodimer pair (**k**, **l**; highlighting by symbols as in **g**). Scale bars are 20 µm

When studying the relationship between galectin architecture and the distribution of staining, experimental conditions for signal generation were deliberately kept constant to ensure comparability. Compared to the WT constellation, the covalent conjugation of Gal-7 CRDs to obtain stable homodimers led to a quantitative increase in signal intensity (Fig. 6a, b, Table 2). The questions on whether and how combining two types of CRD will affect staining profiles is answered next by testing the heterodimers, and Table 2 includes information on the results obtained with WT Gal-1 and the Gal-3 CRD used as donor of a CRD for hybrids. Figure 6c–f indicates the emergence of differences depending on the type of CRD and on the length of the linkers. Pairwise comparisons based on the data given in Table 2 reveal the occurrence of reductions in signal intensity below levels of WT galectins by sequence of CRDs (in the Gal-7/-1 case) and by linker length.

**Table 2:** Distribution and cellular localization of galectin-dependent and Lac-inhibitable staining in sections of fixed adult murine epididymis^a^

Site of staining	Type of protein												
	Gal-7	Gal-7‒Gal-7	Gal-7‒8S‒Gal-7	Gal-7‒Gal-1	Gal-7‒8S‒Gal-1	Gal-1‒Gal-7	Gal-1‒8S‒Gal-7	Gal-1^b^	Gal-7‒Gal-3	Gal-7‒8S‒Gal-3	Gal-3‒Gal-7	Gal-3‒8S‒Gal-7	Gal-3 CRD^c^
Principal cells													
Stereocilia	−/+	+	+	+	−	+	+	++	−	−	−	−/+	−/+
Apical^d^	++	+++	+++	++/+++	−/+	++	+/++	++++	−/+	+/++	−/+	+	++
Supranuclear^d^	++	+++	+++	++/+++	−/+	++	+/++	++++	−/+	+/++	−/+	+	++
Basal^d^	++	+++	+++	++/+++	−/+	++	+/++	++++	−/+	+/++	−/+	+	++
Apical cells	++	+++	+++	++/+++	−/+	++	+/++	++++	−/+	+/++	−/+	+	++
Basal cells	++	+++	+++	+++^e^	++/+++	+++/++++	++++	++++	++++	+ → +++	++/+++	++++	+++/++++
Smooth muscle cells	−	+/++	+	+	+/++	+	++	−	−/+	−/+	−/+	++	−
Connective tissue	−	+/++	+	+	+/++	−/+	+/++	−	−/+	−/+	−/+	+	−/+

^a^Intensity of staining in sections is grouped into the following categories: −, no staining; (+), very weak but above background; +, weak; ++, medium; +++, strong; ++++, very strong

^b/c^From Kutzner et al. 2019^b^ and García Caballero et al. 2020b^c^

^d^Positivity of given regions of cytoplasm

^e^Mostly confined to individual cells

In sections of the adult jejunum, CRD bridging in the case of the homodimer did not affect staining profiles qualitatively, a tendency for increased intensity by covalent conjugation with a minor influence of linker length being apparent (Fig. 6g, h, Table 3). As above, information on binding of Gal-1 and the Gal-3-CRD is presented for comparison in Table 3. The increase in linker length drastically reduced binding, especially of the Gal-7–Gal-1 heterodimer (Fig. 6i, j). This parameter also had an effect on the respective heterodimer of the Gal-7 and Gal-3 CRDs (Fig. 6k, l). Sequence presentation was more a factor for this pair than for heterodimers of Gal-1 and -7 CRDs, in particular when looking at surface enterocytes and lamina propria and goblet cells (Table 3). Obviously, the “where” of a CRD in a heterodimer is relevant.

**Table 3:** Distribution and cellular localization of galectin-dependent and Lac-inhibitable staining in sections of fixed adult murine jejunum^a^

Site of staining	Type of protein												
	Gal-7	Gal-7‒Gal-7	Gal-7‒8S‒Gal-7	Gal-7‒Gal-1	Gal-7‒8S‒Gal-1	Gal-1‒Gal-7	Gal-1‒8S‒Gal-7	Gal-1^b^	Gal-7‒Gal-3	Gal-7‒8S‒Gal-3	Gal-3‒Gal-7	Gal-3‒8S‒Gal-7	Gal-3 CRD^c^
Intestinal villi													
Surface enterocytes													
Brush border	−/+	+	+/++	++	−	−/+	+/++	−/+	+ → +++^d^	−/+	−/+	+	−/+
Apical^e^	++	++/+++	+/++	++	−/+	+/++	−/+	++++	+/++	−/+	−/+ → +	+/++	+
Supranuclear^e^	++	++/+++	+/++	++/+++	−/+	++	−/+ → +	++++	+/++	−/+	−/+ → +	+/++	+
Basal^e^	++	++/+++	+/++	+	−	+	−	++++	−/+ → ++	−/+	−/+	−/+	−/+
Goblet cells													
Theca^f^	+/++	+/++	+/++	+	−	+/++	−	+++	−/+	−/+	+	+	+
Content	−	−	−	−/+	−	−/+	−	−	− → ++++^d^	−	− → ++	−/+	++/+++
Basal	−	−	−	+	−	+	−	+++	−/+	−/+	+	+	+
Lamina propria													
Immune cells	+	+/++	++/+++^g^	−	−	−	−	++	++ → ++++^g^	++++^g^	−/+	−	+ → ++++
Fibroblasts	+	+/++	+/++	+ → +++	+++	++/+++	+++	−/+	++ → ++++^g^	(+)	+++	+/++	−/+
Muscle cells	+	+/++	+/++	+ → +++	−	++	−	−	−	(+)	+++	−/+	−
Endothelial cells	−/+	−/+	−/+	+	−	−	−	−	−	(+)	−/+	−	−
Crypts of Lieberkuhn													
Enterocytes													
Apical^e^	++	++/+++	+/++	++	−	+/++	+	++++	+	−/+	−/+ → +	+	+
Supranuclear^e^	++	++/+++	+/++	++	−	+/++	−/+ → +	++++	+	−/+	−/+ → +	+	+
Basal^e^	++	++	+/++	+/++	−	+	−	++++	−	−/+	−/+	+	+
Goblet cells													
Theca^f^	−	−/+	+/++	++	−	+	−	++	− → +++	−	+	+	++
Content	−	−/+	−	−/+	−	−/+	−	−	− → +++	−	− → ++	−/+	++++
Basal	−	−/+	−	++	−	+	−	++	− → +++	−	+	+	+
Cells at crypts´ base^h^	+ → +++	++++	++++	++++	+ → +++	++++	++++	+++	++++	+ → +++	+++/++++	++++	++++
Muscle layers	++	++	++/+++	(+)/+	−	+/++	+	++	−/(+)	−/(+)	+/++	+/++	−

^a^For grading of staining intensity, please see footnote to Table 2

^b/c^From Kutzner et al. 2019^b^ and García Caballero et al. 2020b^c^

^d^Staining intensity of mucus

^e^Positivity of given regions of cytoplasm

^f^Cup-shaped rim of cytoplasm

^g^Mostly confined to individual cells

^h^Cell population with absorptive cells (enterocytes), enteroendocrine cells, goblet cells (positivity of cytoplasm, no staining of intracellular material in part destined for secretion)

**Fig. 7:**
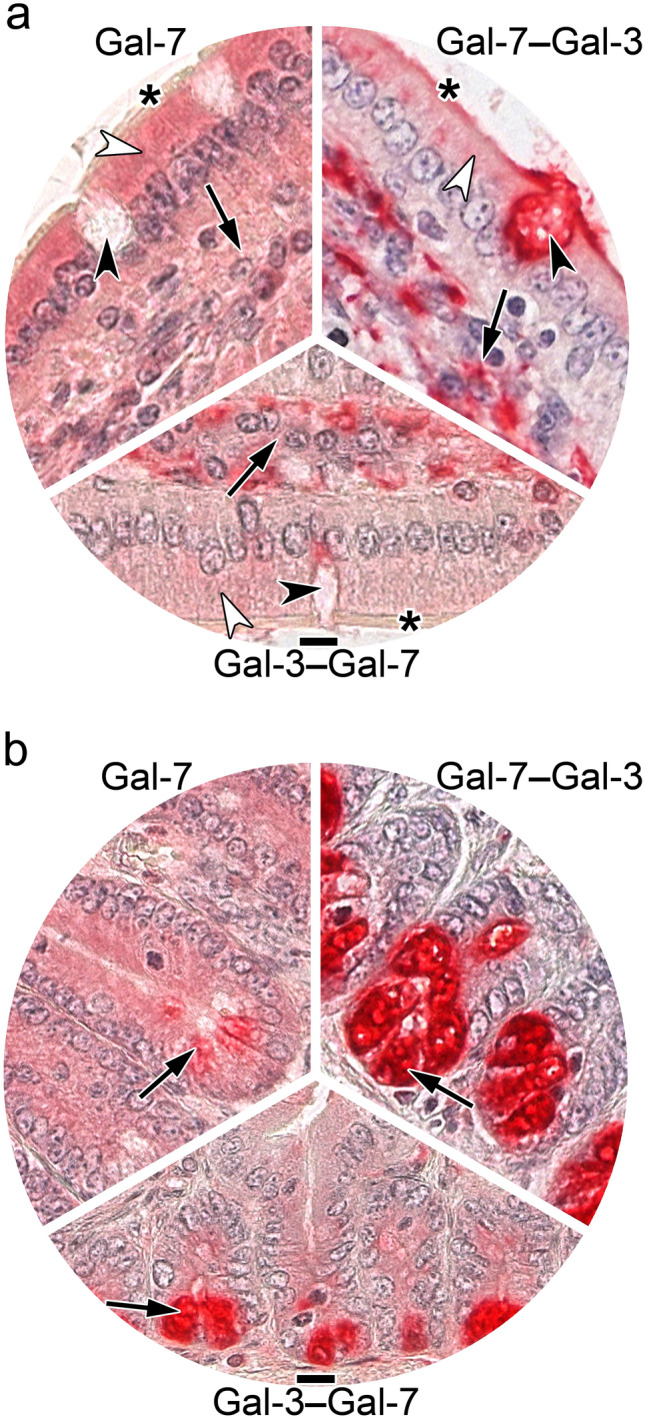
At-a-glance illustration of differences in staining patterns of labeled Gal-7 and the heterodimer pair with sequence permutation, i.e. Gal-7–Gal-3 and Gal-3–Gal-7 in villi (**a**) and the base of crypts (**b**) in sections of fixed murine jejunum. (**a**) Brush border (asterisk), surface enterocytes (white arrowhead), immune cells of the lamina propria (black arrow) and contents of goblet cells (black arrowhead) are highlighted to emphasize distinct aspects, as done in (**b**) for the prominent difference in staining intensity of cells at the base of crypts (arrow; for summary of intensity of staining, please see Table 3). Scale bars are 10 µm

The arrangement of typical photomicrographs of staining profiles (given in Fig. 7) by placing WT Gal-7 in the top-left position and the two Gal-7–Gal-3 CRD-based heterodimers in the other two parts of Fig. 7 underscores the described occurrence of differences in villi (a) and crypts of Lieberkuhn (b). Signal intensity of surface enterocytes and cells of the lamina propria differed between WT Gal-7 and the two Gal-7/-3-based heterodimers as well as in relation to the CRD sequence (Fig. 7a, Table 3). The most prominent and most readily discernible intensity difference concerned cells at the base of crypts (Fig. 7b, Table 3), solidifying the evidence for the impact of design from homo- to heterodimers and of the heterodimer sequence on binding properties to tissue sections.

Having herewith documented binding to disaccharides free in solution by ITC and to cognate sites in tissue sections in lactose-inhibitable manner by galectin histochemistry, the remaining question is whether the apparent bivalency is functional by triggering post-binding effects. The mentioned activity of Gal-7 as growth regulator of neuroblastoma cells furnishes our study with a suitable test system to determine the influence of protein design on a property that depends on counter-receptor cross-linking (lattice formation) and triggering outside-in signaling.

### Cell growth regulation

**Fig. 8:**
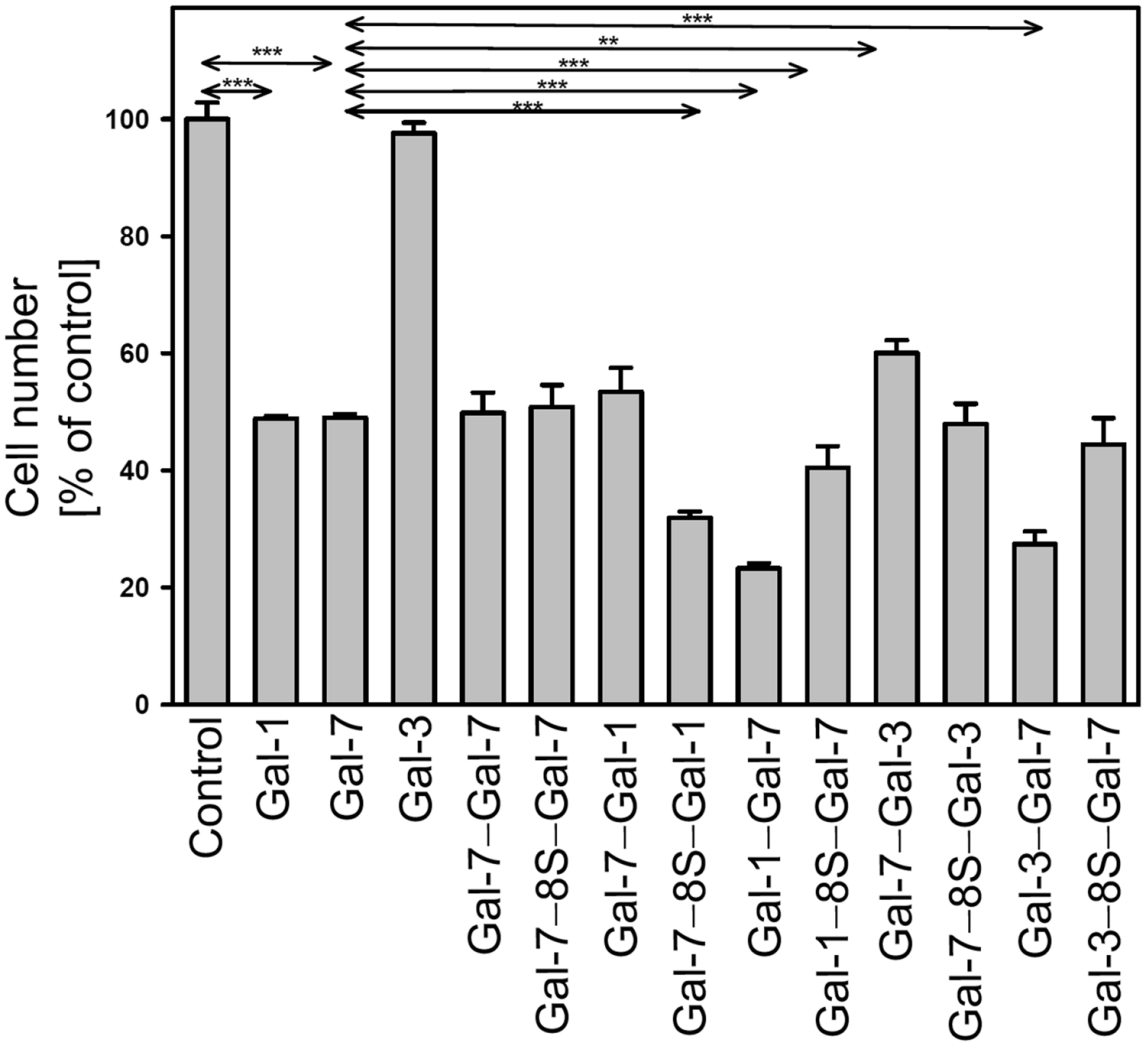
Inhibition of neuroblastoma cell proliferation by wild-type galectins and the panel of engineered variants (**p < 0.01; ***p < 0.001)

The complete panel of variant proteins and the three WT galectins were tested in parallel with aliquots of the same cell suspension per experimental series. Data are presented as graphs in Fig. 8 and by numbers in Supplementary Material, Table S18. Together with the case of the activity of the endogenous effector in the human SK-N-MC neuroblastoma cells, i.e. Gal-1, the previously reported findings that Gal-7 is a potent inhibitor of proliferation (Kopitz et al. 2001, 2003) were confirmed (Fig. 8, left). Gal-3, which lacks the typical bridging capacity of the homodimers to build well-ordered lattices, cannot reduce cell growth (Kopitz et al. 2001), as is also documented in Fig. 8 (left). The first question to be answered is on the impact of covalent conjugation of the Gal-7 CRDs. This artificial way of keeping CRDs together in the two Gal-7 homodimers led to equally potent effectors in this system irrespective of the type of conjugation.

In contrast, the modality of linking CRDs and the arrangement of the positions of the CRDs in heterodimers have a significant impact: the Gal-1(-3)/-7 heterodimer(s) is (are) more potent than WT proteins (at *p* ≤ 0.001) and more active than proteins presenting the Gal-7 CRD first (Fig. 8). Moreover, the presence of the 33-amino-acid linker has context-dependent effects (Fig. 8). Of note, the data show that the Gal-3 CRD becomes active as part of a cross-linking heterodimer (for a checkerboard-style survey of statistical assessments, please see Supplementary Material, Table S19). When using WT Gal-3 as inhibitor in a competition assay for binding of the cellular counter-receptor, i.e. the pentasaccharide of ganglioside GM1, together with the cross-linking homo- and heterodimers (tested at the 10:1 ratio), the expected marked reduction in the cellular response was seen in all cases (Supplementary Material, Fig. S14, Table S18). These results indicate that protein design has the potential to biomedically further improve the already favorable aspects of Gal-7 functionality, here by covalent heterodimer formation with CRDs of Gal-1 or Gal-3 and the Gal-7 CRD at the C terminus.

## Discussion

Combined, the growing evidence for a major role of tissue lectins in cellular (patho)physiology and the remarkable status of the characterization of their structural diversity explain the eminent importance to attribute the modular architecture of galectins to function. Starting the phylogenetic history of a lectin family from an ancestral CRD, sequence divergence and the implementation of distinct types of protein design enable its members to become a toolbox for various duties in cell biology. In addition to elucidating the functionality of WT proteins, changing lectin design in a defined manner opens a new source of relevant information: what chemical modifications have initially achieved, that is to alter the quaternary structure and to relate its status to mitogenicity, is now done rationally on the DNA level, termed lectinology 4.0 (for a review, please see Ludwig et al. 2019b).

Protein engineering supplies the technology to reach the next level, that is to create variants presenting the CRD in diverse structural contexts, more so than naturally available. Their testing is sure to provide instructive insights into why the WT protein best fits into the flow of information, e.g. by coordination of glycoconjugate-lectin recognition with post-binding events. Turning to Gal-1 and our cell model, sensing an upregulation of a counter-receptor as the molecular on-switch for a cellular activity, as is the case for homodimeric Gal-1 and the originally cryptic ganglioside GM1 (obtained by tightly controlled removal of one sialic acid from the ganglioside GD1a glycan) in controlling neuroblastoma proliferation (Kopitz et al. 1998, 2001), in autoimmune regulation (Wang et al. 2009) and in neuritogenesis (Wu et al. 2016), would be lost if Gal-1 were not a homodimer but had a tetrameric CRD presentation. Instead, this design, known to occur in oysters (Tasumi and Vasta 2007), will allow high-affinity binding even at low-level ligand density, which might be helpful in host defense (Kopitz et al. 2017). Delineating the relationship of architecture to bioactivity of a lectin CRD can thus provide an understanding of why certain types of design from a broad, theoretically possible panel were obviously preferred during evolution. Toward this aim, our study initiated protein engineering in the case of Gal-7. We generated stable (permanent) homodimers with the Gal-7 CRD and heterodimers by connecting the Gal-7 CRD with CRDs of Gal-1 or Gal-3, respectively, covalent (direct or linker-involving) conjugation. This study gives reason to the presence of natural Gal-7 as a non-covalently associated homodimer, because its CRD can then under certain conditions become a part of new galectin hybrids.

In our report, we first answered the questions on whether homo- and heterodimers without/with sequence additions to connect CRDs are produced by bacteria and maintain affinity for the canonical sugar (Lac). Recombinant expression in all cases shows that covalently connecting a Gal-7 CRD with a second CRD did not preclude the constructs’ biosynthesis. Purification by affinity chromatography and ITC measurements demonstrate common enthalpy-driven carbohydrate-binding activity of both CRDs in the engineered proteins. The comparatively reduced affinity of Gal-7 for Lac(*N*Ac) relative to Gal-1 and Gal-3 with maintained enthalpic driving force had previously been reported (Ahmad et al. 2002; Dam et al. 2005), setting an internal standard. The crystal structures of human Gal-7 with both sugars illustrate a gain of a single contact for Lac*N*Ac relative to Lac binding (Leonidas et al. 1998). Concerning the core 1 disaccharide of mucin-type *O*-glycans, the detected Gal-7 binding resembles Gal-3 more than Gal-1, as has previously been seen to be the case for binding di-*N*-acetylated Lac (Lacdi*N*Ac; Gal*N*Acβ1,4Glc*N*Ac) (Ludwig et al. 2019a) and selectively accepting internal units instead of the terminal disaccharide in Lac*N*Ac oligomers of *N*- and *O*-glycans as ligand (Ahmad et al. 2002).

As a consequence of Lac binding, the homodimeric status of Gal-7 appeared to be stabilized (Ermakova et al. 2013). So far unique among galectins, the quaternary structure of Gal-7 can be modulated by the isomerization of a prolyl peptide bond (at Pro4): its *cis*-state favors dissociation to monomers at low-micromolar concentrations (Miller et al. 2020). In our histochemical and cell biological assays, the rather similar properties of WT Gal-7 and its two homodimeric variants with covalent bridging between the two CRDs indicate that natural and variant proteins are efficient cross-linkers. The WT protein, yet, offers the added advantage of a regulation of the quaternary structure under certain conditions that is lost by covalent conjugation. This can be of relevance in itself, possibly reflected by observations that the phenotypes of mice either deficient for or overexpressing Gal-7 with destabilized adherens junctions between keratinocytes as hallmark are similar, which points to harm done at this place by a disbalance (Gendronneau et al. 2008, 2015). Moreover, the availability of the monomer in a certain context can facilitate hybrid formation with CRDs of Gal-1 or Gal-3. Respective evidence reported by Miller et al. (2018) had prompted us to design a panel of such heterodimers, and they were obtained by recombinant production in active form. To take the step from measuring carbohydrate-binding activity with free disaccharides to analyzing recognition properties on the level of cells and organs, we were inspired by the successful historical application of lectin histochemistry.

Cellular glycome diversity, for example thoroughly analyzed this way for the intestinal epithelium (Taatjes and Roth 1991), and the presence of various types of cells in tissue sections make them a versatile assay platform for comparative analysis of lectin binding. Analysis of staining profiles will spot differences due to their specificity and design. Binding of the labeled proteins was invariably inhibitable by cognate sugar and not affected by osmolarity controls. In terms of ligand presentation, the bivalent glycocluster was more potent as inhibitor than the tetravalent compound, all dimeric proteins sharing this difference in susceptibility. When examining the staining profiles in detail, it turned out that the order of CRD arrangement (Gal-7–Gal-X vs Gal-X–Gal-7) and the nature of the spacing between CRDs (no extra amino acid or the Gal-8S linker peptide) can matter, and the data from our proliferation assays corroborate this conclusion. These results intimate the experimental perspective to let CRDs switch places in tandem-repeat-type galectins and examine the activity of WT protein and its variant. Likewise, dissecting the activity of hybrids in cell systems, in which counter-receptor(s) and elicited post-binding mechanisms differ between WT galectins, apoptosis induction in activated T cells by Gal-1 or Gal-7 via disparate caspase activation profiles offering a suggestion for a suited cell system (Sturm et al. 2004), is of interest.

Although not directly comparable with the intention of our study design, the importance of the position of a CRD in a dimer is a phenomenon first seen in the case of galectins when testing hybrids formed with a CRD of the tandem-repeat-type Gal-9 (N- or C-terminal) and the Gal-1 CRD: the prominent status of the C-terminal CRD of Gal-9 for galectin-induced T cell death was shown in this setting (Bi et al. 2008). In the case of Gal-8, testing of N–N/C–C constructs unveiled the dominant role of its N-terminal CRD for platelet activation (Romaniuk et al. 2010). Concerning the aspect of linker length for Gal-8, the following observation underlines the potential of this parameter: when inserting a (Gly)_6_ linker instead of its natural linker into human Gal-8, adhesive and signaling capacity tested on CHO cells was impaired (Levy et al. 2006; please see also next paragraph). These case studies emphasize, along with our work, the need to be mindful of each detail of galectin design and to consider this aspect for engineering of variants with the aim of applications.

Our respective results with the engineered hybrids have a cell biological dimension. So far, the study of galectins has mostly focused on separate analysis of each protein. It is now advancing to include the consideration of networking between WT galectins (Habermann et al. 2021). Intriguingly, the possibility for hybrids to occur transiently in cells furnishes access to reversible heterobifunctionality built by non-covalently associated CRDs. Like the mentioned cases of tandem-repeat-type Gal-8 and Gal-9, Gal-4 with its two different CRDs and its binding to the sulfatide headgroup (via the N-terminal CRD) and *N*-glycans of glycoproteins (in apical or axonal transport and delivery) sets an instructive example for how effectively two CRDs in a galectin cooperate (Stechly et al. 2009; Velasco et al. 2013; Murphy et al. 2021). Modulating the length of a linker in this case and for Gal-8 attenuates cell binding and bridging and introduces a sensor for reacting to ligand presence at low density (Kopitz et al. 2012; Zhang et al. 2015; Xiao et al. 2018). The revelation of Gal-7 (-1)-mediated association of radioiodinated Gal-3 CRD to the neuroblastoma cell surface had provided first evidence for the gain of a new property of a CRD by hybrid generation (Miller et al. 2018). Since the Gal-7 CRD appears to engage in interactions with glycan and protein counter-receptors, shown to be operative for instance in proper E-cadherin dynamics in adherens junctions (Advedissian et al. 2017), as the Gal-3 CRD has been described to make contact with glycans and the chemokine CXCL12 via its S- and F-faces (Eckardt et al. 2020), binding partners for a bridging process can also include certain proteins. The case studies on Gal-3, Gal-8 and Gal-9 in autophagy and vesicle repair (Hong et al. 2018) or on the eye lens-specific member of the galectin family called GRIFIN (García Caballero et al. 2019, 2020c) underscore bispecificity to be common among galectins: building heterodimers is suited to expanding the range of cognate pairing to new combinations. Their availability enables the purification of counter-receptors by affinity chromatography using the hybrids described here as specific baits and product characterization comparatively for WT and hybrid galectins.

Since their generation in situ will naturally depend on the co-expression of the building-block CRDs, the presence and level of heterodimers are then subject to tight spatiotemporal control. Immunohistochemical colocalization that verifies co-expression at the cellular level and excludes inverse shifts between components, seen for example in head and neck tumor progression (Saussez et al. 2008), will be the prerequisite for selecting cellular models as study objects to appraise the assumed biorelevance. Since galectins lack a signal sequence and are exported on non-classical routes from cells (Hughes 1999; Sato 2018; Kutzner et al. 2020), examining an influence of hybrid formation on intracellular activity and secretion will be a further topic of research. Of course, the detected indication for enhanced bioactivity in our growth assays provides incentive for testing stable heterodimers in settings where two natural galectins are known to cooperate, e.g. in wound healing (Cao et al. 2002; Dvoránková et al. 2011; Gál et al. 2021). Suppression of tumor growth by Gal-7 gene transfer (Ueda et al. 2004) may further benefit from firmly locking the quaternary structure.

In summary, the non-covalently associated Gal-7 homodimer presents no major difference in our assays relative to stable (covalently linked) variants. However, that it is the natural Gal-7 version does not appear to be coincidental. Physiologically, this type of the homodimer appears to undergo dynamic monomerization, apparently favored at low concentration by a *cis/trans*-isomerization of the proline(Pro) 4 prolyl peptide bond (Miller et al. 2020). This ability would be completely lost by the artificial covalent connection. When then available as monomer, the possibility of forming hybrids lets new combinations of CRDs arise locally within the galectin panel. In principle, the prototype design endows cells with more functional opportunities than a permanent homodimer status. Our results reveal changes in the activity of the respective variants depending on the nature of the linker and on the spatial order of the individual CRDs, remarkably an increased growth-regulatory effect. In addition to directing efforts to further explore heterodimer activity and to detect their presence and characterize functions in vivo, their potential for enhanced bioactivity or for a gain of new functions compared to WT galectins may well warrant attention when considering biomedical applications of custom-made variants of human galectins.

## Supplementary Information

Below is the link to the electronic supplementary material.

Supplementary file1 (DOCX 76 KB)

Supplementary file2 (PPTX 6174 KB)

## Data Availability

Data will be made available on reasonable request.
