# Supplementary material for: Imitating evolution’s tinkering by protein engineering reveals extension of human galectin-7 activity
Source: Histochem Cell Biol. Author manuscript; Available in PMC 2021 Sep 24. (PMC8460509; doi:10.1007/s00418-021-02004-w)
Supplement: 1722537_Sup_Tables [file NIHMS1722537-supplement-1722537_Sup_Tables.docx]

**Table S1** Oligonucleotides

| **Primer** | **Sequence** |
| --- | --- |
| hGal-7_NdeI_FOR | 5'-GCACATATGTCCAACGTCCCCCAC-3' |
| hGal-7_HindIII_REV | 5'-CGTAAGCTTTCAGAAGATCCTCACGGAG-3' |
| hGal-1–hGal-7_FOR | 5'-CAAATGTGTGGCCTTTGACTCCAACGTCCCCCACAAG-3' |
| hGal-1–hGal-7_REV | 5'-CTTGTGGGGGACGTTGGAGTCAAAGGCCACACATTTG-3' |
| 8S–hGal-7_FOR | 5'-CTGGCACGCCCCAGCTTTCCAACGTCCCCCACAAG-3' |
| 8S–hGal-7_REV | 5'-CTTGTGGGGGACGTTGGAAAGCTGGGGCGTGCCAG-3' |
| hGal-7–8S_FOR | 5'-GACTCCGTGAGGATCTTCTTTAGCTTCAGCTCGGAC-3' |
| hGal-7–8S_REV | 5'-GTCCGAGCTGAAGCTAAAGAAGATCCTCACGGAGTC-3' |
| hGal-7–hGal-1_FOR | 5'-GACTCCGTGAGGATCTTCGCTTGTGGTCTGGTCGC-3' |
| hGal-7–hGal-1_REV | 5'-GCGACCAGACCACAAGCGAAGATCCTCACGGAGTC-3' |
| hGal-7–hGal-7_FOR | 5'-GACTCCGTGAGGATCTTCTCCAACGTCCCCCACAAG-3' |
| hGal-7–hGal-7_REV | 5'-CTTGTGGGGGACGTTGGAGAAGATCCTCACGGAGTC-3' |
| hGal-1–8S_FOR | 5'-ctggcacgccccagcttgcttgtggtctggtcgc-3' |
| hGal-1–8S_REV | 5'-gtaagtccgagctgaagctaaagtcaaaggccacacatttg-3' |
| hGal-3–8S_FOR | 5'-GTGCTTCATATACCATGATAtttagcttcagctcggac-3' |
| hGal-3–8S_REV | 5'-gtccgagctgaagctaaaTATCATGGTATATGAAGCAC-3' |
| 8S–hGal-1_FOR | 5'-caaatgtgtggcctttgactttagcttcagctcggacttac-3' |
| 8S–hGal-1_REV | 5'-gcgaccagaccacaagcaagctggggcgtgccag-3' |
| 8S–hGal-3_FOR | 5'-ctggcacgccccagcttGGCGCCCCTGCTGGGCC-3' |
| 8S–hGal-3_REV | 5'-GGCCCAGCAGGGGCGCCaagctggggcgtgccag-3' |
| hGal-1_Nde_For | 5'-gcacatatggcttgtggtctggtc-3' |
| hGal-1_Hind_Rev | 5'-cgtaagctttcagtcaaaggccacac-3' |
| hGal-3CRD­_Nde_For | 5'-gctcatatgGGCGCCCCTGCTGGGC-3' |
| hGal-3_Hind_Rev | 5'-cgtaagctttcagtcaaaggccacac-3' |

**Table S2** Conditions of expressions and yield of recombinant galectins

| **Protein** | **Bacterial Strain** | **Expression Vector** | **Expression Temp. °C** | **IPTG (µM)** | **Yield (mg/L)** |
| --- | --- | --- | --- | --- | --- |
| Gal-7 | BL21 (DE3) pLysS | pGEMEX-1 | 22 °C | 100 µM | 175 ± 1.41 (n=2) |
| Gal-7–Gal-7 | Rosetta (DE3) pLysS | pET24a | 22 °C | 100 µM | 122.48 ± 90.36 (n=4) |
| Gal-7–8S–Gal-7 | Rosetta (DE3) pLysS | pET24a | 22 °C | 100 µM | 50.84 ± 39.87 (n=5) |
| Gal-7–Gal-1 | Rosetta (DE3) pLysS | pET24a | 22 °C | 75 µM | 130.52 ±34.61 (n=3) |
| Gal-7–8S–Gal-1 | Rosetta (DE3) pLysS | pET24a | 22 °C | 500 µM | 16.14± 6.81 (n=5) |
| Gal-1–Gal-7 | BL21 (DE3) pLysS | pGEMEX-1 | 37 °C | 75 µM | 126.1 ± 46.19 (n=3) |
| Gal-1–8S–Gal-7 | BL21 (DE3) pLysS | pGEMEX-1 | 37 °C | 75 µM | 104,6 ± 14.71 (n=2) |
| Gal-7–Gal-3 | Rosetta (DE3) pLysS | pET24a | 22 °C | 100 µM | 192.42 ± 139.7 (n=2) |
| Gal-7–8S–Gal-3 | Rosetta (DE3) pLysS | pET24a | 22 °C | 100 µM | 14.81 ± 6.88 (n=4) |
| Gal-3–Gal-7 | BL21 (DE3) pLysS | pGEMEX-1 | 22 °C | 100 µM | 145.23 ± 91.54 (n=3) |
| Gal-3–8S–Gal-7 | BL21 (DE3) pLysS | pGEMEX-1 | 22 °C | 100 µM | 45.74 ± 9.28 (n=2) |

**Table S3** Tested concentration ranges in galectin histochemistry and values for best signal-to-background ratio (optimal concentrations)

| **Type of protein** | **Concentration range** | **Optimal concentration** |
| --- | --- | --- |
| Gal-7 | 0.5 – 12.0 µg/mL | 4.0 µg/mL^a^; 8.0 µg/mL^b^ |
| Gal-7–Gal-7 | 0.5 – 8.0 µg/mL | 4.0 µg/mL |
| Gal-7–8S–Gal-7 | 0.5 – 8.0 µg/mL | 4.0 µg/mL^a^; 2.0 µg/mL^b^ |
| Gal-7–Gal-1 | 0.125 – 1.0 µg/mL | 0.25 µg/mL |
| Gal-7–8S–Gal-1 | 0.125 – 1.0 µg/mL | 0.25 µg/mL |
| Gal-1–Gal-7 | 0.0625 – 2.0 µg/mL | 1.0 µg/mL^a^; 0.5 µg/mL^b^ |
| Gal-1–8S–Gal-7 | 0.0625 – 1.0 µg/mL | 0.25 µg/mL^a^; 0.125 µg/mL^b^ |
| Gal-1^c^ | 0.0625 – 4.0 µg/mL | 0.5 µg/mL^a^; 0.25 µg/mL^b^ |
| Gal-7–Gal-3 | 0.125 – 1.0 µg/mL | 0.5 µg/mL^a^; 0.25 µg/mL^b^ |
| Gal-7–8S–Gal-3 | 0.5 – 8.0 µg/mL | 2.0 µg/mL^a^; 0.5 µg/mL^b^ |
| Gal-3–Gal-7 | 0.0625 – 1.0 µg/mL | 0.25 µg/mL^a^; 0.5 µg/mL^b^ |
| Gal-3-8S–Gal-7 | 0.125 – 1.0 µg/mL | 0.25 µg/mL |
| Gal-3 CRD^d^ | 2.0 – 32.0 µg/mL | 16.0 µg/mL^a^; 12.0 µg/mL^b^ |

^a^sections of fixed adult murine epididymis

^b^sections of fixed adult murine jejunum

^c^Kutzner et al. 2019

^d^Garcia Caballero et al. 2020b

**Table S4** Peptide mass fingerprinting of Gal-7–8S–Gal-7 by MALDI-TOF-MS. The list of detected peptides with their calculated (calc) and experimentally

measured (exp) mass values

| **Sequence** | **[MH]^+^ (mono) calc** | **[MH]^+^ (mono) exp** |
| --- | --- | --- |
| EQGSWGR | 819.4 | 819.5 |
| GPGVPFQR | 857.5 | 857.6 |
| HRLPLAR | 862.5 | 862.6 |
| GLVPPNASR | 910.5 | 910.6 |
| LDTSEVVFNSK | 1238.6 | 1238.8 |
| AVVGDAQYHHFR | 1399.7 | 1399.9 |
| SSLPEGIRPGTVLR | 1481.8 | 1482.0 |
| LVEVGGDVQLDSVR | 1485.8 | 1486.0 |
| AVVGDAQYHHFRHR | 1692.8 | 1693.0 |
| GQPFEVLIIASDDGFK | 1736.0 | 1736.1 |
| LVEVGGDVQLDSVRIF | 1745.9 | 1746.1 |
| ENVPKSGTPQLSNVPHK | 1832.0 | 1832.0 |
| VRLVEVGGDVQLDSVRIF | 2001.1 | 2001.4 |
| LDTSEVVFNSKEQGSWGR | 2039.0 | 2039.2 |
| FHVNLLCGEEQGSDAALHFNPR | 2454.2 | 2454.3 |
| FHVNLLCGEEQGSDAALHFNPR^1^ | 2511.2 | 2511.4 |
| IFFSFSSDLQSTQASSLELTEISR | 2693.3 | 2693.5 |
| GQPFEVLIIASDDGFKAVVGDAQYHHFR | 3116.6 | 3116.6 |

^1^carbamidomethylation at C

**Table S5** Peptide mass fingerprinting of Gal-7–Gal-7 by MALDI-TOF-MS. The list of detected peptides with their calculated (calc) and experimentally

measured (exp) mass values

| **Sequence** | **[MH]^+^ (mono) calc** | **[MH]^+^ (mono) exp** |
| --- | --- | --- |
| EQGSWGR | 819.4 | 819.5 |
| GPGVPFQR | 857.5 | 857.6 |
| HRLPLAR | 862.5 | 862.7 |
| GLVPPNASR | 910.5 | 910.6 |
| IFSNVPHK | 941.5 | 941.6 |
| IRGLVPPNASR | 1179.7 | 1179.7 |
| EQGSWGREER | 1233.6 | 1233.7 |
| LDTSEVVFNSK | 1238.6 | 1238.8 |
| EERGPGVPFQR | 1271.6 | 1271.8 |
| AVVGDAQYHHFR | 1399.7 | 1399.9 |
| SSLPEGIRPGTVLR | 1481.8 | 1482.0 |
| LVEVGGDVQLDSVR | 1485.8 | 1486.0 |
| GQPFEVLIIASDDGFK | 1735.9 | 1736.1 |
| LDTSEVVFNSKEQGSWGR | 2039.0 | 2039.2 |
| LDTSEVVFNSKEQGSWGREER | 2453.2 | 2453.4 |
| FHVNLLCGEEQGSDAALHFNPR | 2454.2 | 2454.4 |
| FHVNLLCGEEQGSDAALHFNPR^1^ | 2511.2 | 2511.4 |
| VRLVEVGGDVQLDSVRIFSNVPHK | 2663.5 | 2663.4 |

^1^carbamidomethylation at C

**Table S6** Peptide mass fingerprinting of Gal-7–Gal-1 by MALDI-TOF-MS. The list of detected peptides with their calculated (calc) and experimentally measured (exp) mass values

| **Sequence** | **[MH]^+^ (mono) calc** | **[MH]^+^ (mono) exp** |
| --- | --- | --- |
| EQGSWGR | 819.4 | 819.5 |
| GPGVPFQR | 857.5 | 857.6 |
| HRLPLAR | 862.5 | 862.7 |
| SFVLNLGK | 877.5 | 877.6 |
| GLVPPNASR | 910.5 | 910.7 |
| LPDGYEFK | 968.5 | 968.6 |
| VRGEVAPDAK | 1041.6 | 1041.7 |
| DGGAWGTEQR | 1076.5 | 1076.7 |
| IRGLVPPNASR | 1179.7 | 1179.8 |
| EQGSWGREER | 1233.6 | 1233.7 |
| LDTSEVVFNSK | 1238.6 | 1238.8 |
| EERGPGVPFQR | 1271.6 | 1271.8 |
| AVVGDAQYHHFR | 1399.7 | 1399.9 |
| DSNNLCLHFNPR | 1429.7 | 1429.8 |
| SSLPEGIRPGTVLR | 1481.8 | 1482.0 |
| DSNNLCLHFNPR^1^ | 1486.7 | 1486.8 |
| FNAHGDANTIVCNSK | 1647.8 | 1648.0 |
| GQPFEVLIIASDDGFK | 1736.0 | 1736.1 |
| VRLVEVGGDVQLDSVR | 1741.0 | 1741.2 |
| LNLEAINYMAADGDFK | 1784.9 | 1785.1 |
| LDTSEVVFNSKEQGSWGREER | 2039.0 | 2039.2 |
| SNVPHKSSLPEGIRPGTVLR | 2144.2 | 2144.4 |
| IFACGLVASNLNLKPGECLR^1^ | 2175.1 | 2175.3 |
| IFACGLVASNLNLKPGECLR^1,1^ | 2232.2 | 2232.4 |
| FHVNLLCGEEQGSDAALHFNPR | 2454.2 | 2454.4 |
| FHVNLLCGEEQGSDAALHFNPR^1^ | 2511.2 | 2511.5 |
| FNAHGDANTIVCNSKDGGAWGTEQR^1^ | 2705.2 | 2705.5 |

^1^carbamidomethylation at C

**Table S7** Peptide mass fingerprinting of Gal-7–8S–Gal-1 by MALDI-TOF-MS. The list of detected peptides with their calculated (calc) and experimentally measured (exp) mass values

| **Sequence** | **[MH]^+^ (mono) calc** | **[MH]^+^ (mono) exp** |
| --- | --- | --- |
| PGECLR^1^ | 731.4 | 731.4 |
| EQGSWGR | 819.4 | 819.4 |
| GPGVPFQR | 857.5 | 857.5 |
| HRLPLAR | 862.5 | 862.6 |
| SFVLNLGK | 877.5 | 877.6 |
| GLVPPNASR | 910.5 | 910.6 |
| VRGEVAPDAK | 1041.6 | 1041.7 |
| DGGAWGTEQR | 1076.5 | 1076.6 |
| IRGLVPPNASR | 1179.7 | 1179.7 |
| EQGSWGREER | 1233.6 | 1233.7 |
| LDTSEVVFNSK | 1238.6 | 1238.8 |
| EERGPGVPFQR | 1271.6 | 1271.8 |
| AVVGDAQYHHFR | 1399.7 | 1399.8 |
| DSNNLCLHFNPR | 1429.7 | 1429.7 |
| SSLPEGIRPGTVLR | 1481.8 | 1482.0 |
| DSNNLCLHFNPR^1^ | 1486.7 | 1486.8 |
| FNAHGDANTIVCNSK | 1647.8 | 1647.9 |
| GQPFEVLIIASDDGFK | 1736.0 | 1736.0 |
| VRLVEVGGDVQLDSVR | 1741.0 | 1741.1 |
| SFVLNLGKDSNNLCLHFNPR^1^ | 2345.2 | 2345.3 |
| LNEISKLGISGDIDLTSASYTMI | 2441.2 | 2441.4 |
| FHVNLLCGEEQGSDAALHFNPR | 2454.2 | 2454.3 |
| FHVNLLCGEEQGSDAALHFNPR^1^ | 2511.2 | 2511.4 |
| FPNRLNLEAINYMAADGDFKIK | 2540.3 | 2540.5 |
| SGTPQLACGLVASNLNLKPGECLR^1,1^ | 2555.3 | 2555.6 |
| LNLEAINYMAADGDFKIKCVAFD^2^ | 2577.2 | 2577.6 |
| IFFSFSSDLQSTQASSLELTEISR | 2693.3 | 2693.7 |
| EAVFPFQPGSVAEVCITFDQANLTVK^1^ | 2867.4 | 2867.8 |

^1^carbamidomethylation at C

^2^oxidation at M

**Table S8** Peptide mass fingerprinting of Gal-1–Gal-7 by MALDI-TOF-MS. The list of detected peptides with their calculated (calc) and experimentally

measured (exp) mass values

| **Sequence** | **[MH]^+^ (mono) calc** | **[MH]^+^ (mono) exp** |
| --- | --- | --- |
| EQGSWGR | 819.4 | 819.4 |
| GPGVPFQR | 857.5 | 857.5 |
| HRLPLAR | 862.5 | 862.6 |
| SFVLNLGK | 877.5 | 877.5 |
| GLVPPNASR | 910.5 | 910.5 |
| LPDGYEFK | 968.5 | 968.5 |
| VRGEVAPDAK | 1041.6 | 1041.6 |
| DGGAWGTEQR | 1076.5 | 1076.5 |
| IRGLVPPNASR | 1179.7 | 1179.7 |
| EQGSWGREER | 1233.6 | 1233.6 |
| LDTSEVVFNSK | 1238.6 | 1238.7 |
| CVAFDSNVPHK^1^ | 1273.6 | 1273.7 |
| AVVGDAQYHHFR | 1399.7 | 1399.7 |
| DSNNLCLHFNPR | 1429.7 | 1429.7 |
| SSLPEGIRPGTVLR | 1481.8 | 1482.0 |
| DSNNLCLHFNPR^1^ | 1486.7 | 1486.9 |
| FNAHGDANTIVCNSK | 1647.8 | 1647.8 |
| GQPFEVLIIASDDGFK | 1736.0 | 1735.9 |
| VRLVEVGGDVQLDSVR | 1741.0 | 1741.0 |
| LNLEAINYMAADGDFK | 1784.9 | 1785.0 |
| ACGLVASNLNLKPGECLR^1^ | 1915.0 | 1915.1 |
| ACGLVASNLNLKPGECLR^1,1^ | 1972.0 | 1972.2 |
| EQGSWGREERGPGVPFQR | 2072.0 | 2072.1 |
| LDTSEVVFNSKEQGSWGREER | 2453.2 | 2453.2 |
| FHVNLLCGEEQGSDAALHFNPR^1^ | 2511.2 | 2511.3 |
| FNAHGDANTIVCNSKDGGAWGTEQR^1^ | 2705.2 | 2705.3 |
| EAVFPFQPGSVAEVCITFDQANLTVK^1^ | 2867.4 | 2867.4 |
| LPDGYEFKFPNRLNLEAINYMAADGDFK^2^ | 3264.6 | 3264.8 |

^1^carbamidomethylation at C

^2^oxidation at M

**Table S9** Peptide mass fingerprinting of Gal-1–8S–Gal-7 by MALDI-TOF-MS. The list of detected peptides with their calculated (calc) and experimentally measured (exp) mass values

| **Sequence** | **[MH]^+^ (mono) calc** | **[MH]^+^ (mono) exp** |
| --- | --- | --- |
| PGECLR^1^ | 731.4 | 731.4 |
| EQGSWGR | 819.4 | 819.5 |
| GPGVPFQR | 857.5 | 857.6 |
| HRLPLAR | 862.5 | 862.7 |
| SFVLNLGK | 877.5 | 877.6 |
| GLVPPNASR | 910.5 | 910.6 |
| LPDGYEFK | 968.5 | 968.6 |
| VRGEVAPDAK | 1041.6 | 1041.7 |
| DGGAWGTEQR | 1076.5 | 1076.6 |
| EQGSWGREER | 1233.6 | 1233.7 |
| LDTSEVVFNSK | 1238.6 | 1238.7 |
| SGTPQLSNVPHK | 1264.7 | 1264.7 |
| EERGPGVPFQR | 1271.6 | 1271.8 |
| AVVGDAQYHHFR | 1399.7 | 1399.8 |
| SSLPEGIRPGTVLR | 1481.8 | 1482.0 |
| DSNNLCLHFNPR^1^ | 1486.7 | 1486.9 |
| FNAHGDANTIVCNSK | 1647.8 | 1647.9 |
| GQPFEVLIIASDDGFK | 1736.0 | 1736.0 |
| LVEVGGDVQLDSVRIF | 1745.9 | 1746.0 |
| LNLEAINYMAADGDFK | 1784.9 | 1785.0 |
| ACGLVASNLNLKPGECLR^1.1^ | 1972.0 | 1972.2 |
| SFVLNLGKDSNNLCLHFNPR^1^ | 2345.2 | 2345.4 |
| FHVNLLCGEEQGSDAALHFNPR^1^ | 2511.2 | 2511.5 |
| FNAHGDANTIVCNSKDGGAWGTEQR^1^ | 2705.2 | 2705.5 |
| EAVFPFQPGSVAEVCITFDQANLTVK^1^ | 2867.4 | 2867.8 |
| CVAFDFSFSSDLQSTQASSLELTEISR^1^ | 3025.4 | 3025.9 |

^1^carbamidomethylation at C

**Table S10** Peptide mass fingerprinting of Gal-7–Gal-3 by MALDI-TOF-MS. The list of detected peptides with their calculated (calc) and experimentally

measured (exp) mass values

| **Sequence** | **[MH]^+^ (mono) calc** | **[MH]^+^ (mono) exp** |
| --- | --- | --- |
| LNEISK | 703.4 | 703.5 |
| FNENNR | 793.4 | 793.5 |
| EQGSWGR | 819.4 | 819.5 |
| GPGVPFQR | 857.5 | 857.5 |
| HRLPLAR | 862.5 | 862.6 |
| LDNNWGR | 874.4 | 874.5 |
| GLVPPNASR | 910.5 | 910.6 |
| RVIVCNTK | 932.5 | 932.6 |
| FNENNRR | 949.5 | 949.6 |
| IRGLVPPNASR | 1179.7 | 1179.8 |
| EQGSWGREER | 1233.6 | 1233.7 |
| LDTSEVVFNSK | 1238.6 | 1238.8 |
| GNDVAFHFNPR | 1273.6 | 1273.8 |
| LDNNWGREER | 1288.6 | 1288.8 |
| IQVLVEPDHFK | 1324.7 | 1324.9 |
| AVVGDAQYHHFR | 1399.7 | 1399.9 |
| SSLPEGIRPGTVLR | 1481.8 | 1482.0 |
| LVEVGGDVQLDSVR | 1485.8 | 1486.0 |
| QSVFPFESGKPFK | 1497.8 | 1498.0 |
| MLITILGTVKPNANR | 1641.0 | 1641.2 |
| VAVNDAHLLQYNHR | 1649.9 | 1650.1 |
| GQPFEVLIIASDDGFK | 1736.0 | 1736.1 |
| VRLVEVGGDVQLDSVR | 1741.0 | 1741.2 |
| LDTSEVVFNSKEQGSWGREER | 2039.0 | 2039.2 |
| EQGSWGREERGPGVPFQR | 2072.0 | 2072.2 |
| IFGAPAGPLIVPYNLPLPGGVVPR | 2414.4 | 2414.6 |
| LDTSEVVFNSKEQGSWGREER | 2453.2 | 2453.4 |
| FHVNLLCGEEQGSDAALHFNPR^1^ | 2511.2 | 2511.4 |
| GQPFEVLIIASDDGFKAVVGDAQYHHFR | 3116.6 | 3116.8 |

^1^carbamidomethylation at C

**Table S11** Peptide mass fingerprinting of Gal-7–8S–Gal-3 by MALDI-TOF-MS. The list of detected peptides with their calculated (calc) and experimentally

measured (exp) mass values

| **Sequence** | **[MH]^+^ (mono) calc** | **[MH]^+^ (mono) exp** |
| --- | --- | --- |
| LNEISK | 703.4 | 703.5 |
| FNENNR | 793.4 | 793.5 |
| EQGSWGR | 819.4 | 819.5 |
| GPGVPFQR | 857.5 | 857.5 |
| HRLPLAR | 862.5 | 862.6 |
| LDNNWGR | 874.4 | 874.6 |
| GLVPPNASR | 910.5 | 910.7 |
| RVIVCNTK | 932.5 | 932.6 |
| FNENNRR | 949.5 | 949.6 |
| RVIVCNTK^1^ | 989.6 | 989.7 |
| IRGLVPPNASR | 1179.7 | 1179.8 |
| EQGSWGREER | 1233.6 | 1233.7 |
| LDTSEVVFNSK | 1238.6 | 1238.8 |
| GNDVAFHFNPR | 1273.6 | 1273.8 |
| LDNNWGREER | 1288.6 | 1288.8 |
| IQVLVEPDHFK | 1324.7 | 1324.9 |
| AVVGDAQYHHFR | 1399.7 | 1399.9 |
| SSLPEGIRPGTVLR | 1481.8 | 1482.0 |
| LVEVGGDVQLDSVR | 1485.8 | 1486.0 |
| QSVFPFESGKPFK | 1497.8 | 1498.0 |
| MLITILGTVKPNANR | 1641.0 | 1641.2 |
| VAVNDAHLLQYNHR | 1649.9 | 1650.1 |
| FNENNRRVIVCNTK | 1706.9 | 1707.1 |
| GQPFEVLIIASDDGFK | 1736.0 | 1736.1 |
| VRLVEVGGDVQLDSVR | 1741.0 | 1741.1 |
| RVIVCNTKLDNNWGR | 1787.9 | 1788.0 |
| LDTSEVVFNSKEQGSWGREER | 2039.0 | 2039.1 |
| SNVPHKSSLPEGIRPGTVLR | 2144.2 | 2144.4 |
| LNEISKLGISGDIDLTSASYTMI | 2441.2 | 2441.5 |
| FHVNLLCGEEQGSDAALHFNPR | 2454.2 | 2454.4 |
| FHVNLLCGEEQGSDAALHFNPR^1^ | 2511.2 | 2511.4 |
| IFFSFSSDLQSTQASSLELTEISR | 2693.3 | 2693.6 |
| SGTPQLGAPAGPLIVPYNLPLPGGVVPR | 2737.5 | 2737.8 |
| GQPFEVLIIASDDGFKAVVGDAQYHHFR | 3116.6 | 3117.0 |
| IFFSFSSDLQSTQASSLELTEISRENVPK  ^1^carbamidomethylation at C | 3260.6 | 3261.2 |

**Table S12** Peptide mass fingerprinting of Gal-3–Gal-7 by MALDI-TOF-MS. The list of detected peptides with their calculated (calc) and experimentally

measured (exp) mass values

| **Sequence** | **[MH]^+^ (mono) calc** | **[MH]^+^ (mono) exp** |
| --- | --- | --- |
| LNEISK | 703.4 | 703.4 |
| FNENNR | 793.4 | 793.4 |
| EQGSWGR | 819.4 | 819.4 |
| GPGVPFQR | 857.5 | 857.5 |
| HRLPLAR | 862.5 | 862.6 |
| LDNNWGR | 874.4 | 874.5 |
| GLVPPNASR | 910.5 | 910.6 |
| RVIVCNTK | 932.5 | 932.5 |
| FNENNRR | 949.5 | 949.5 |
| EQGSWGREER | 1233.6 | 1233.6 |
| LDTSEVVFNSK | 1238.6 | 1238.7 |
| GNDVAFHFNPR | 1273.6 | 1273.7 |
| LDNNWGREER | 1288.6 | 1288.7 |
| IQVLVEPDHFK | 1324.7 | 1324.8 |
| AVVGDAQYHHFR | 1399.7 | 1399.8 |
| SSLPEGIRPGTVLR | 1481.8 | 1482.0 |
| LVEVGGDVQLDSVR | 1485.8 | 1485.9 |
| QSVFPFESGKPFK | 1497.8 | 1497.9 |
| MLITILGTVKPNANR | 1641.0 | 1641.1 |
| VAVNDAHLLQYNHR | 1649.9 | 1650.0 |
| FNENNRRVIVCNTK | 1706.9 | 1707.0 |
| GQPFEVLIIASDDGFK | 1736.0 | 1736.0 |
| LVEVGGDVQLDSVRIF | 1745.9 | 1746.1 |
| LDTSEVVFNSKEQGSWGREER | 2039.0 | 2039.1 |
| GAPAGPLIVPYNLPLPGGVVPR | 2154.2 | 2154.4 |
| LGISGDIDLTSASYTMISNVPHK | 2419.2 | 2419.4 |
| FHVNLLCGEEQGSDAALHFNPR | 2454.2 | 2454.3 |
| FHVNLLCGEEQGSDAALHFNPR^1^ | 2511.2 | 2511.5 |
| GQPFEVLIIASDDGFKAVVGDAQYHHFR | 3116.6 | 3116.9 |
| GAPAGPLIVPYNLPLPGGVVPRMLITILGTVK | 3223.9 | 3223.7 |

^1^carbamidomethylation at C

**Table S13** Peptide mass fingerprinting of Gal-3–8S–Gal-7 by MALDI-TOF-MS. The list of detected peptides with their calculated (calc) and experimentally measured (exp) mass values

| **Sequence** | **[MH]^+^ (mono) calc** | **[MH]^+^ (mono) exp** |
| --- | --- | --- |
| LNEISK | 703.4 | 703.5 |
| FNENNR | 793.4 | 793.5 |
| EQGSWGR | 819.4 | 819.5 |
| VIVCNTK^1^ | 833.5 | 833.6 |
| GPGVPFQR | 857.5 | 857.6 |
| HRLPLAR | 862.5 | 862.6 |
| LDNNWGR | 874.4 | 874.5 |
| GLVPPNASR | 910.5 | 910.6 |
| RVIVCNTK | 932.5 | 932.6 |
| FNENNRR | 949.5 | 949.6 |
| RVIVCNTK^1^ | 989.6 | 989.7 |
| IRGLVPPNASR | 1179.7 | 1179.8 |
| EQGSWGREER | 1233.6 | 1233.7 |
| LDTSEVVFNSK | 1238.6 | 1238.7 |
| SGTPQLSNVPHK | 1264.7 | 1264.8 |
| GNDVAFHFNPR | 1273.6 | 1273.8 |
| LDNNWGREER | 1288.6 | 1288.8 |
| IQVLVEPDHFK | 1324.7 | 1324.9 |
| AVVGDAQYHHFR | 1399.7 | 1399.9 |
| SSLPEGIRPGTVLR | 1481.8 | 1482.0 |
| LVEVGGDVQLDSVR | 1485.8 | 1486.0 |
| QSVFPFESGKPFK | 1497.8 | 1498.0 |
| MLITILGTVKPNANR | 1641.0 | 1641.1 |
| VAVNDAHLLQYNHR | 1649.9 | 1650.0 |
| GQPFEVLIIASDDGFK | 1736.0 | 1736.0 |
| VRLVEVGGDVQLDSVR | 1741.0 | 1741.1 |
| LVEVGGDVQLDSVRIF | 1745.9 | 1746.1 |
| VRLVEVGGDVQLDSVRIF | 2001.1 | 2001.3 |
| LDTSEVVFNSKEQGSWGREER | 2039.0 | 2039.2 |
| EQGSWGREERGPGVPFQR | 2072.0 | 2072.1 |
| VIVCNTKLDNNWGREER^1^ | 2103.0 | 2103.2 |
| SNVPHKSSLPEGIRPGTVLR | 2144.2 | 2154.4 |
| LDTSEVVFNSKEQGSWGREER | 2453.2 | 2453.3 |
| FHVNLLCGEEQGSDAALHFNPR^1^ | 2511.2 | 2511.4 |
| SGTPQLSNVPHKSSLPEGIRPGTVLR | 2727.5 | 2727.7 |
| GQPFEVLIIASDDGFKAVVGDAQYHHFR | 3116.6 | 3116.9 |

^1^carbamidomethylation at C

**Table S14** Summary of thermodynamics of association Lac (6.0 mM) to human Gal-7 and its homo- and heterodimers with Gal-1 and Gal-3 at 25 °C calculated using the one-set-of-sites binding model

| Lectin | [Cell]  (μM) | *n* | *K*_a_  (x10^4^ M^-1^) | -Δ*G*  (kcal/mol) | -Δ*H*  (kcal/mol) | -*T*Δ*S*  (kcal/mol) | *K*_d_  (μM) |
| --- | --- | --- | --- | --- | --- | --- | --- |
| Gal-7 | 120 | 1.74 | 0.31 | 4.76 | 11.20 ± 0.224 | 6.45 | 323 ± 5.70 |
| Gal-7–Gal-7^a^ | 22 | 2.00 | 0.25 | 4.64 | 6.70 ± 0.386 | 2.05 | 396 ± 3.28 |
|  | 15 | 2.00 fixed | 0.23 | 4.58 | 9.42 ± 0.170 | 4.85 | 443 ± 10.3 |
| Gal-7–8S–Gal-7 | 25 | 1.94 | 0.31 | 4.83 | 6.62 ± 1.32 | 1.86 | 325 ± 14.9 |
| Gal-7–Gal-1 | 33 | 2.00 | 0.29 | 4.73 | 9.99 ± 0.727 | 5.25 | 340 ± 6.88 |
|  | 25 | 2.00 fixed | 0.27 | 4.69 | 13.10 ± 0.261 | 8.45 | 364 ± 8.65 |
| Gal-1–Gal-7 | 30 | 2.02 | 0.32 | 4.79 | 9.91 ± 0.973 | 5.11 | 308 ± 8.90 |
| Gal-1–8S–Gal-7 | 65 | 1.99 | 0.33 | 4.80 | 12.30 ± 1.06 | 7.53 | 305 ± 15.3 |
| Gal-1^b^ | 87 | 0.33 | 4.80 | 3.79 | 8.59 ± 0.109 | 1.90 | 307 ± 5.53 |
| Gal-7–Gal-3 | 40 | 2.00 | 0.58 | 5.14 | 13.90 ± 1.19 | 8.78 | 170 ± 7.78 |
|  | 50 | 2.00 fixed | 0.68 | 5.23 | 10.40 ± 0.285 | 5.15 | 147 ± 7.04 |
| Gal-7–8S–Gal-3 | 25 | 1.99 | 1.01 | 5.46 | 7.02 ± 0.450 | 1.55 | 99 ± 2.96 |
| Gal-3–Gal-7 | 37 | 2.00 | 0.56 | 5.12 | 16.70 ± 1.12 | 11.50 | 179 ± 5.92 |
|  | 50 | 2.00 fixed | 0.62 | 5.18 | 12.30 ± 0.182 | 7.09 | 161 ± 5.42 |
| Gal-3–8S–Gal-7 | 50 | 2.00 | 0.50 | 5.05 | 16.60 ± 1.08 | 11.6 | 200 ± 7.83 |
|  | 55 | 2.00 fixed | 0.57 | 5.13 | 14.10 ± 0.219 | 8.94 | 174 ± 4.49 |
| Gal-3 ^c^ | 88.5 | 0.85 | 0.87 | 5.38 | 13.10 ± 0.541 | 7.73 | 115 ± 2.99 |

^a^Concentration of ligand is 10 mM

^b/c^ From Kutzner et al. 2019^b^ and García Caballero et al. 2020b^c^

**Table S15** Summary of thermodynamics of association Galβ1,3Gal*N*Ac to human Gal-7 and Gal-3^c^ at 25 °C calculated using the one-set-of-sites binding model

| Lectin | [Cell]  (μM) | *n* | *K*_a_  (x 10^4^ M^-1^) | -Δ*G*  (kcal/mol) | -Δ*H*  (kcal/mol) | -*T*Δ*S*  (kcal/mol) | *K*_d_  (μM) |
| --- | --- | --- | --- | --- | --- | --- | --- |
| Gal-7^a^ | 205 | 2.02 | 0.08 | 3.96 | 6.13 ± 0.868 | 2.17 | 1250 ± 126 |
|  | 150 | 2.00 fixed | 0.06 | 3.81 | 9.67 ± 0.688 | 5.86 | 1610 ± 106 |
| Gal-3^b,c^ | 110 | 1.02 | 0.45 | 4.99 | 9.95 ± 1.37 | 4.99 | 222 ± 18.5 |
| Gal-3 CRD^b,c^ | 90 | 0.95 | 0.38 | 4.88 | 7.64 ± 0.95 | 2.75 | 264 ± 13.7 |

^a/b^Concentration of Galβ1,3Gal*N*Ac is 6 mM^a^ and 10 mM^b^

^c^García Caballero et al. 2020b

**Table S16** Summary of thermodynamics of association Lac*N*Ac (6.0 mM) to heterodimers of human Gal-7 with CRDs of Gal-1 or -3

at 25 °C calculated using the sequential binding model

| Lectin | [Cell]  (μM) | *n* | *K*_a1_/*K*_a2_  (x10^4^ M^-1^) | -Δ*G*_1_/- Δ*G*_2_  (kcal/mol) | -Δ*H*_1_/- Δ*H*_2_  (kcal/mol) | -*T*Δ*S*_1_/-*T*Δ*S*_2_  (kcal/mol) | *K*_d1_/ *K*_d2_  (μM) |
| --- | --- | --- | --- | --- | --- | --- | --- |
| Gal-1–Gal-7 | 90 | 2.00 | 1.74/0.33 | 5.80/4.80 | 11.00 ± 0.151  15.60 ± 0.347 | 5.20/10.80 | 57 ± 1.08/  303 ± 0.012 |
| Gal-1–8S–Gal-7 | 60 | 2.00 | 1.44/0.31 | 5.73/4.80 | 13.60 ± 0.247  16.90 ± 0.531 | 7.87/12.10 | 69 ± 1.65/  313 ± 0.030 |
| Gal-7–Gal-3 | 35 | 2.00 | 1.51/0.33 | 5.70/4.77 | 23.10 ± 0.935  13.20 ± 0.671 | 17.40/8.43 | 66 ± 0.005/  299 ± 50.4 |
| Gal-7–8S–Gal-3 | 20 | 2.00 | 8.33/0.84 | 6.73/5.35 | 7.58 ± 0.098  13.70 ± 0.344 | 0.85/8.35 | 12 ± 0.488/  119 ± 4.99 |
| Gal-3–Gal-7 | 40 | 2.00 | 2.50/0.56 | 6.03/5.20 | 15.20 ± 0.101  20.30 ± 0.396 | 9.17/15.10 | 40 ± 0.545/  177 ± 2.61 |
| Gal-3–8S–Gal-7 | 55 | 2.00 | 11.1/0.55 | 6.91/5.10 | 9.44 ± 0.174  24.10 ± 0.198 | 2.53/19.00 | 9 ± 0.0002/  179 ± 5.61 |

**Table S17** Summary of thermodynamics of association Galβ1,3Gal*N*Ac to heterodimers of human Gal-7 with the CRD of Gal-3 at 25 °C calculated using the sequential binding model

| Lectin | [Cell]  (μM) | *n* | *K*_a1_/*K*_a2_  (x10^4^ M^-1^) | -Δ*G*_1_/- Δ*G*_2_  (kcal/mol) | -Δ*H*_1_/- Δ*H*_2_  (kcal/mol) | -*T*Δ*S*_1_/-*T*Δ*S*_2_  (kcal/mol) | *K*_d1_/ *K*_d2_  (μM) |
| --- | --- | --- | --- | --- | --- | --- | --- |
| Gal-3–Gal-7^a^ | 79 | 2.00 | 0.85/0.08 | 5.36/4.04 | 5.12 ± 0.316  14.00 ± 1.57 | -0.24/9.96 | 117 ± 10.50/  1180 ± 0.129 |
| Gal-3–8S–Gal-7^b^ | 55 | 2.00 | 0.34/0.07 | 4.90/3.94 | 14.90 ± 0.138  10.60 ± 0.728 | 10.00/6.66 | 292 ± 4.25/  1380 ± 20.0 |

^a/b^Concentration of Galβ1,3Gal*N*Ac is 6 mM^a^ or 10 mM^b^

**Table S18** Percentage of cell number of neuroblastoma cells

| Galectin | Cell number % of control (±S.D.) | |
| --- | --- | --- |
|  | No addition of Gal-3 | 10-fold excess of Gal-3 |
| Gal-1 | 48.9 (± 0.5) | 81.2 (± 0.9) |
| Gal-7 | 49.0 (± 0.6) | 82.9 (± 0.6) |
| Gal-3 | 97.6 (± 1.8) |  |
| Gal-7–Gal-7 | 49.8 (± 3.5) | 83.1 (± 3.2) |
| Gal-7–8S–Gal-7 | 50.8 (± 3.8) | 84.5 (± 3.4) |
| Gal-7–Gal-1 | 53.4 (± 4.1) | 86.4 (± 2.8) |
| Gal-7–8S–Gal-1 | 31.9*** (± 1.1) | 77.2 (± 1.4) |
| Gal-1–Gal-7 | 23.3*** (± 0.9) | 74.8 (± 2.1) |
| Gal-1–8S–Gal-7 | 40.5** (± 3.6) | 78.0 (±3.3) |
| Gal-7–Gal-3 | 60.1** (± 2.1) | 89.7 (±2.9) |
| Gal-7–8S–Gal-3 | 47.9 (± 3.4) | 81.3 (±3.2) |
| Gal-3–Gal-7 | 27.4*** (± 2.2) | 76,9 (±2.0) |
| Gal-3–8S–Gal-7 | 44.4 (± 3.5) | 80.2 (± 4.1) |

Results are the mean of 4 independent measurements ± S.D.

t-test vs Gal-7: *p < 0.05; **p < 0.01; ***p < 0.001

**Table S19** Complete assessment of levels of statistical significance in pairwise processing of data on growth regulation by WT and variant galectins

|  | **Control** | **Gal-1** | **Gal-7** | **Gal-3** | **Gal-7−Gal-7** | **Gal-7−8S−Gal-7** | **Gal-7−Gal-1** | **Gal-7−8S−Gal-1** | **Gal-1-Gal-7** | **Gal-1−8S−Gal-7** | **Gal-7−Gal-3** | **Gal-7−8S−Gal-3** | **Gal-3−Gal-7** | **Gal-3−8S−Gal-7** |
| --- | --- | --- | --- | --- | --- | --- | --- | --- | --- | --- | --- | --- | --- | --- |
| **Control** |  | *** | *** | **n.s**. | *** | *** | *** | *** | *** | *** | *** | *** | *** | *** |
| **Gal-1** | *** |  | **n.s.** | *** | **n.s.** | **n.s.** | * | *** | *** | *** | ** | **n.s.** | *** | **n.s.** |
| **Gal-7** | *** | **n.s.** |  | *** | **n.s.** | **n.s.** | * | *** | *** | *** | ** | **n.s.** | *** | **n.s.** |
| **Gal-3** | **n.s.** | *** | *** |  | *** | *** | *** | *** | *** | *** | *** | *** | *** | *** |
| **Gal-7−Gal-7** | *** | **n.s.** | **n.s.** | *** |  | **n.s.** | **n.s.** | ** | ** | * | * | **n.s.** | ** | **n.s.** |
| **Gal-7−8S−Gal-7** | *** | **n.s.** | **n.s.** | *** | **n.s.** |  | **n.s.** | ** | *** | ** | * | **n.s.** | ** | **n.s.** |
| **Gal-7−Gal-1** | *** | * | * | *** | **n.s.** | **n.s.** |  | *** | *** | ** | * | * | *** | * |
| **Gal-7−8S−Gal-1** | *** | *** | *** | *** | ** | ** | *** |  | *** | ** | *** | ** | * | ** |
| **Gal-1-Gal-7** | *** | *** | *** | *** | ** | *** | *** | *** |  | ** | *** | *** | * | ** |
| **Gal-1−8S−Gal-7** | *** | *** | *** | *** | * | ** | ** | ** | ** |  | ** | ** | ** | **n.s.** |
| **Gal-7−Gal-3** | *** | ** | ** | *** | * | * | * | *** | *** | ** |  | ** | *** | ** |
| **Gal-7−8S−Gal-3** | *** | **n.s.** | **n.s.** | *** | **n.s.** | **n.s.** | * | ** | *** | ** | ** |  | ** | **n.s.** |
| **Gal-3−Gal-7** | *** | *** | *** | *** | ** | ** | *** | * | * | ** | *** | ** |  | *** |
| **Gal-3−8S−Gal-7** | *** | **n.s.** | **n.s.** | *** | **n.s.** | **n.s.** | * | ** | ** | **n.s.** | ** | **n.s.** | *** |  |

***** p < 0.05; ****** p < 0.01; ******* p < 0.001; n.s.: not significant; graphical data illustration given in Fig. 8
