# Supplementary material for: Imitating evolution’s tinkering by protein engineering reveals extension of human galectin-7 activity
Source: Histochem Cell Biol. Author manuscript; Available in PMC 2021 Sep 24. (PMC8460509; doi:10.1007/s00418-021-02004-w)
Supplement: 1722537_supp_fig [file NIHMS1722537-supplement-1722537_supp_fig.pptx]

## Slide 1
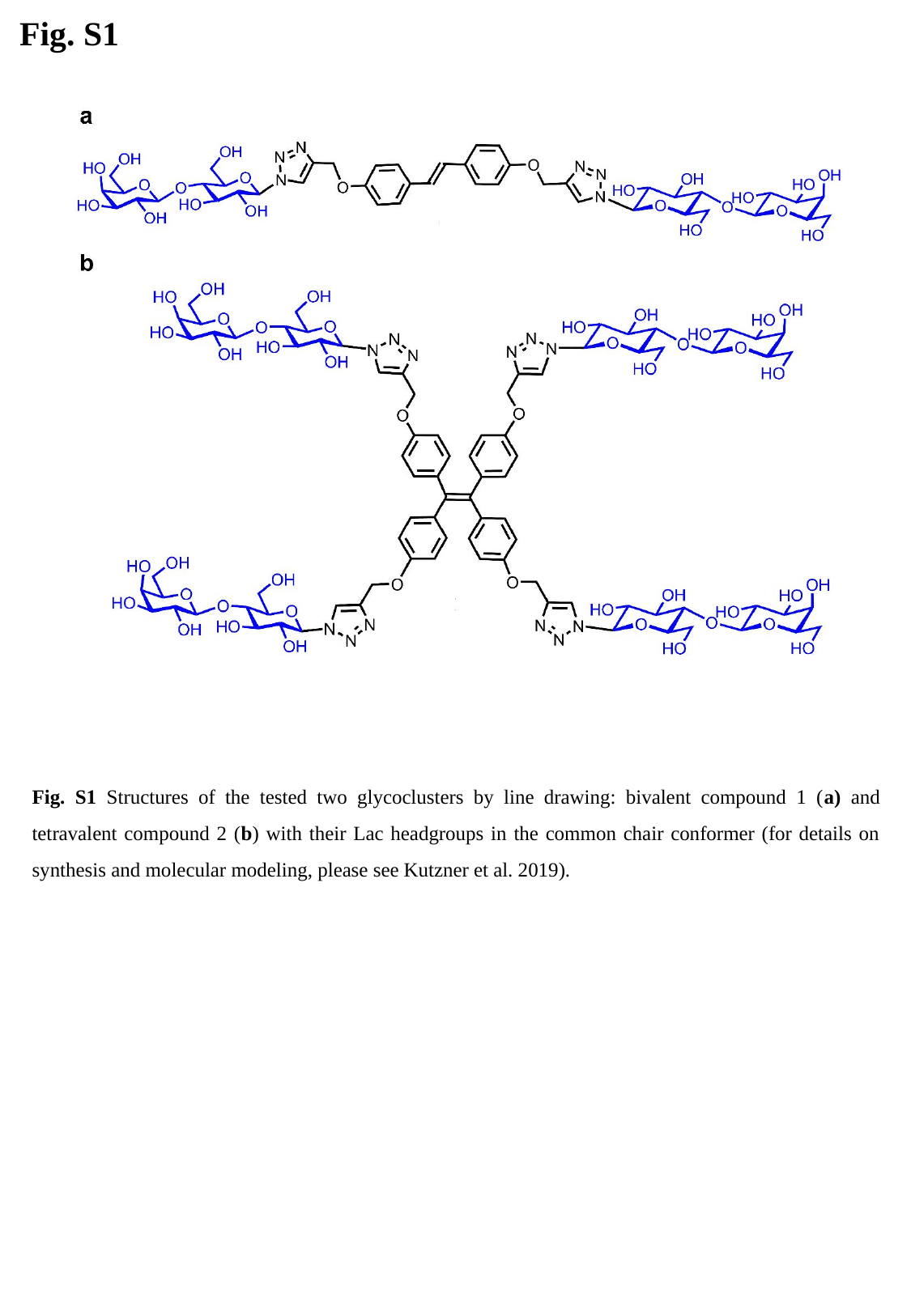

Fig. S1
Fig. S1 Structures of the tested two glycoclusters by line drawing: bivalent compound 1 (a) and tetravalent compound 2 (b) with their Lac headgroups in the common chair conformer (for details on synthesis and molecular modeling, please see Kutzner et al. 2019).

## Slide 2
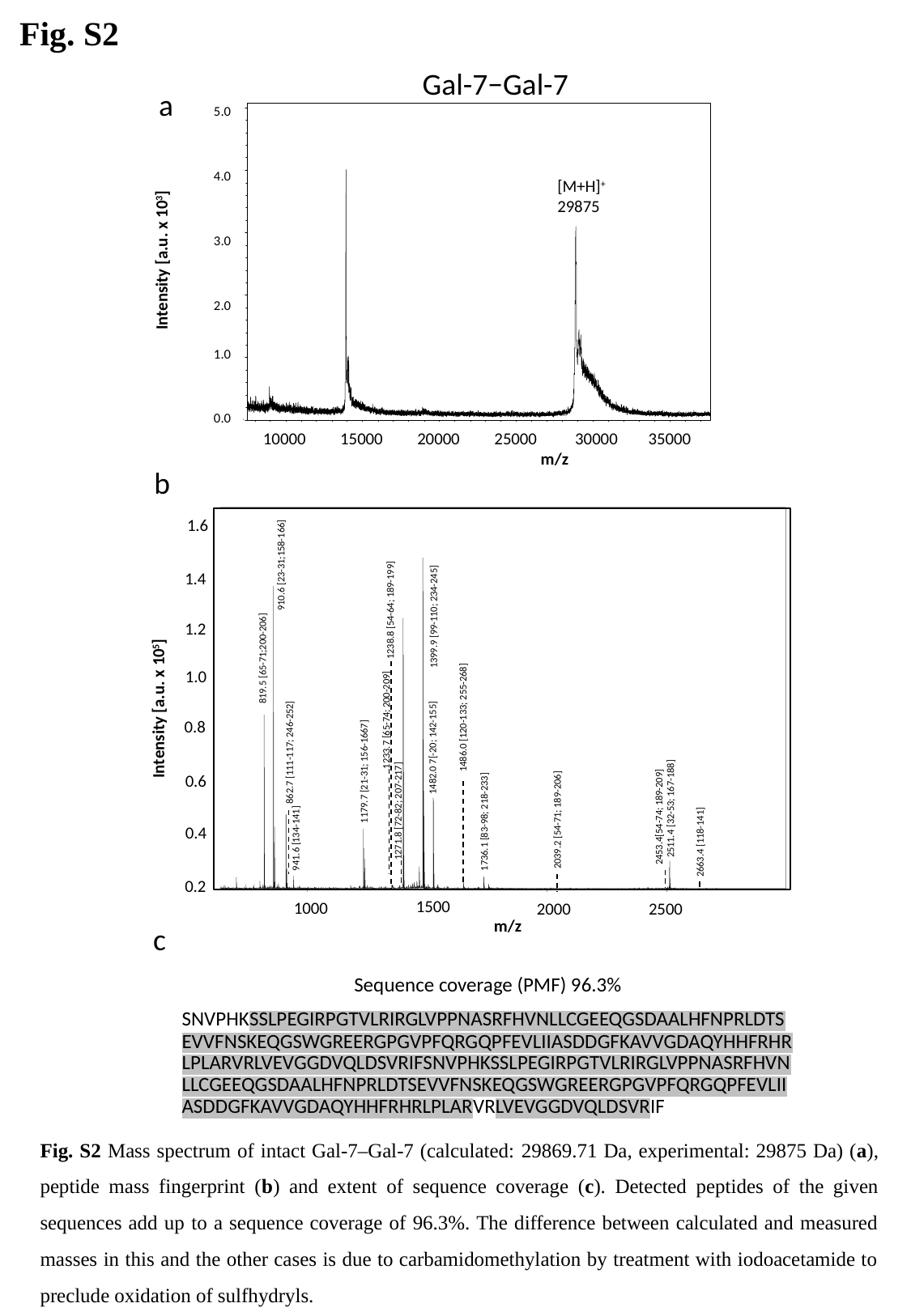

Fig. S2
Gal-7−Gal-7
a
5.0
4.0
3.0
2.0
1.0
0.0
[M+H]+
29875
Intensity [a.u. x 103]
 15000 20000 25000 30000 35000
		 m/z
b
1.6
910.6 [23-31;158-166]
1238.8 [54-64; 189-199]
1399.9 [99-110; 234-245]
1.4
819.5 [65-71;200-206]
1.2
1486.0 [120-133; 255-268]
1.0
Intensity [a.u. x 105]
1233.7 [65-74; 200-209]
862.7 [111-117; 246-252]
0.8
1482.0 7[-20; 142-155]
1179.7 [21-31; 156-1667]
2511.4 [32-53; 167-188]
1271.8 [72-82; 207-217]
2453.4[54-74; 189-209]
0.6
2039.2 [54-71; 189-206]
1736.1 [83-98; 218-233]
941.6 [134-141]
2663.4 [118-141]
0.4
0.2
1500
1000
2500
2000
m/z
c
Sequence coverage (PMF) 96.3%
SNVPHKSSLPEGIRPGTVLRIRGLVPPNASRFHVNLLCGEEQGSDAALHFNPRLDTSEVVFNSKEQGSWGREERGPGVPFQRGQPFEVLIIASDDGFKAVVGDAQYHHFRHRLPLARVRLVEVGGDVQLDSVRIFSNVPHKSSLPEGIRPGTVLRIRGLVPPNASRFHVNLLCGEEQGSDAALHFNPRLDTSEVVFNSKEQGSWGREERGPGVPFQRGQPFEVLIIASDDGFKAVVGDAQYHHFRHRLPLARVRLVEVGGDVQLDSVRIF
Fig. S2 Mass spectrum of intact Gal-7–Gal-7 (calculated: 29869.71 Da, experimental: 29875 Da) (a), peptide mass fingerprint (b) and extent of sequence coverage (c). Detected peptides of the given sequences add up to a sequence coverage of 96.3%. The difference between calculated and measured masses in this and the other cases is due to carbamidomethylation by treatment with iodoacetamide to preclude oxidation of sulfhydryls.

## Slide 3
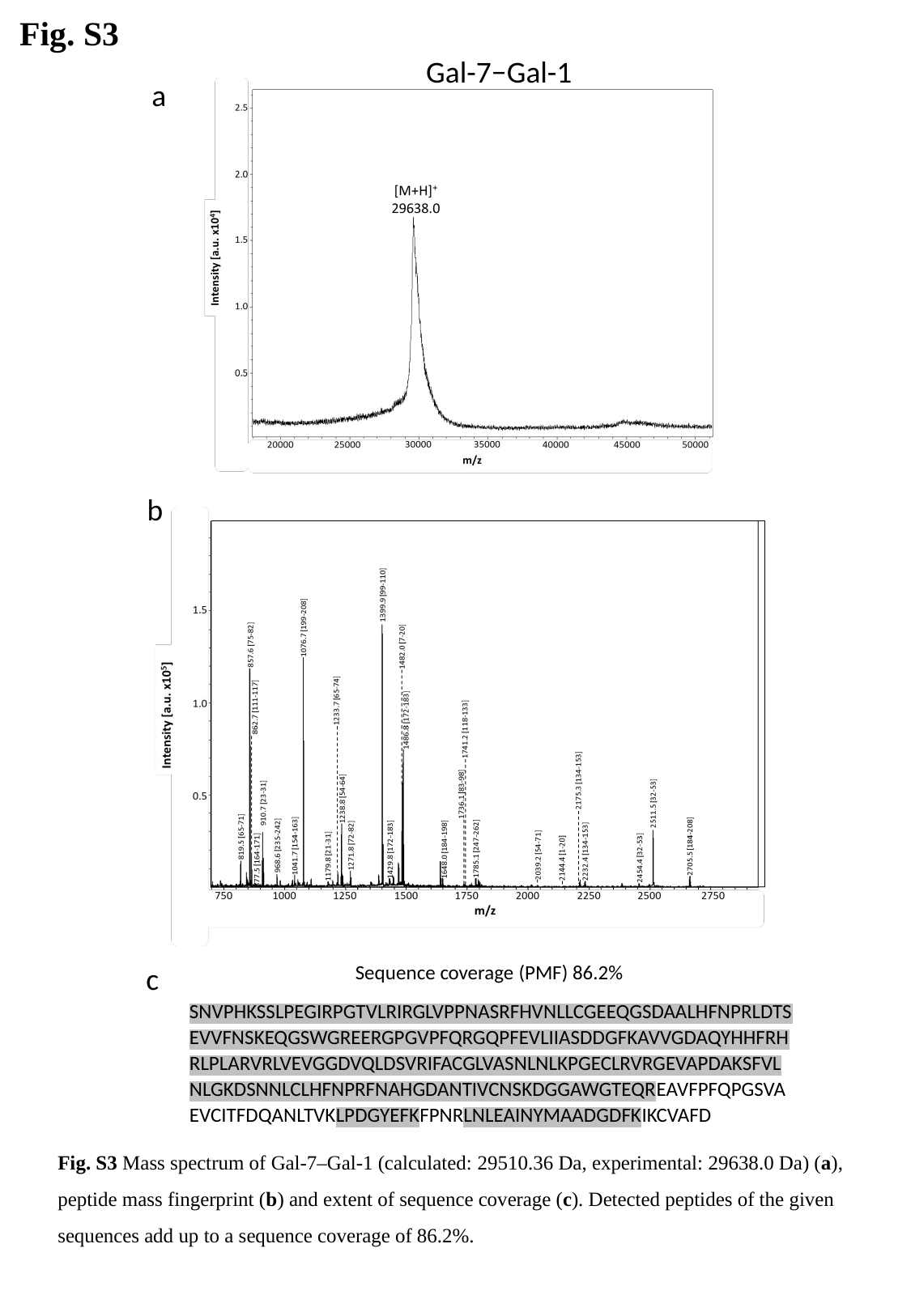

Fig. S3
Gal-7−Gal-1
a
b
c
Sequence coverage (PMF) 86.2%
SNVPHKSSLPEGIRPGTVLRIRGLVPPNASRFHVNLLCGEEQGSDAALHFNPRLDTSEVVFNSKEQGSWGREERGPGVPFQRGQPFEVLIIASDDGFKAVVGDAQYHHFRHRLPLARVRLVEVGGDVQLDSVRIFACGLVASNLNLKPGECLRVRGEVAPDAKSFVLNLGKDSNNLCLHFNPRFNAHGDANTIVCNSKDGGAWGTEQREAVFPFQPGSVAEVCITFDQANLTVKLPDGYEFKFPNRLNLEAINYMAADGDFKIKCVAFD
Fig. S3 Mass spectrum of Gal-7–Gal-1 (calculated: 29510.36 Da, experimental: 29638.0 Da) (a), peptide mass fingerprint (b) and extent of sequence coverage (c). Detected peptides of the given sequences add up to a sequence coverage of 86.2%.

## Slide 4
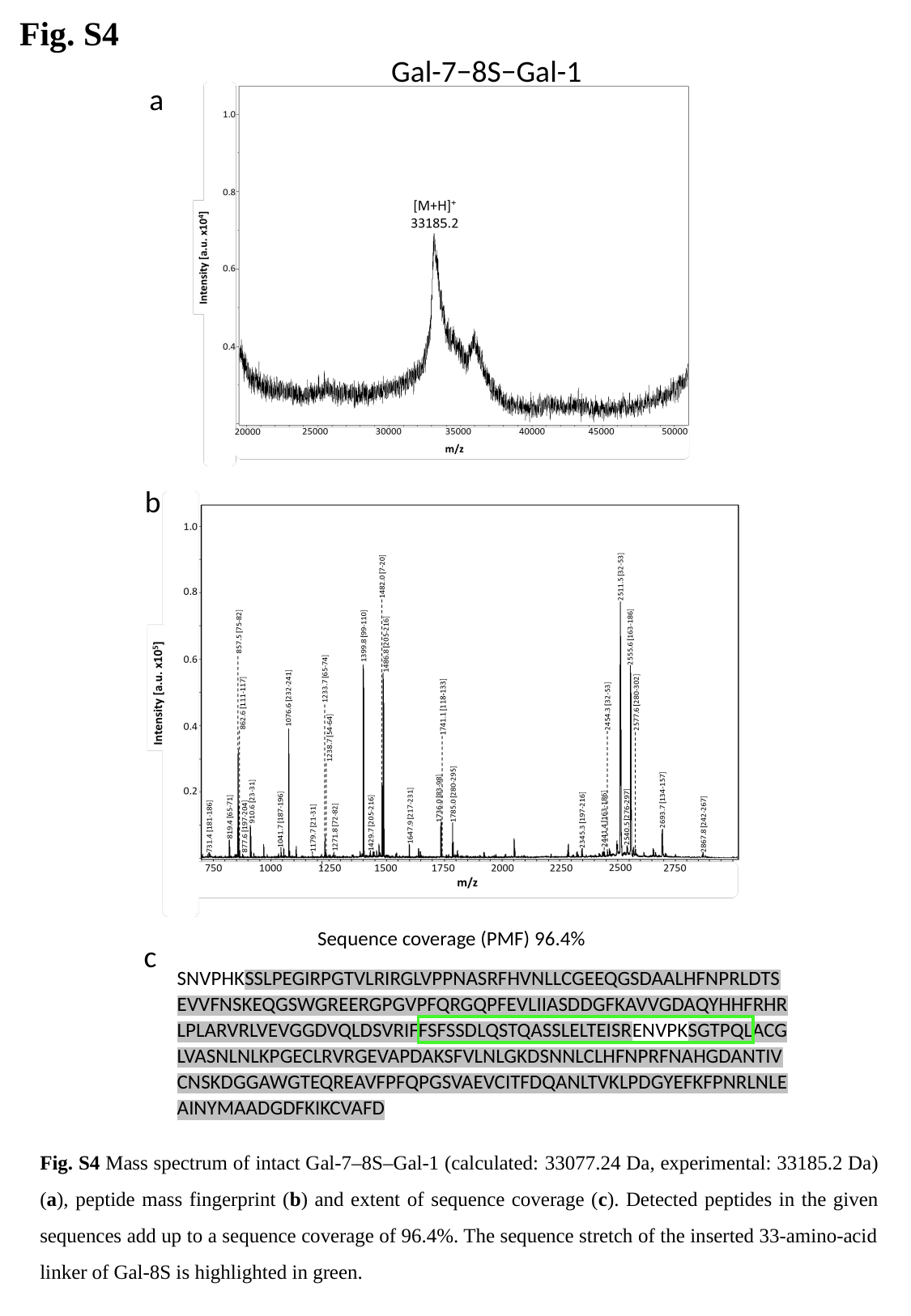

Fig. S4
Gal-7−8S−Gal-1
a
b
Sequence coverage (PMF) 96.4%
c
SNVPHKSSLPEGIRPGTVLRIRGLVPPNASRFHVNLLCGEEQGSDAALHFNPRLDTSEVVFNSKEQGSWGREERGPGVPFQRGQPFEVLIIASDDGFKAVVGDAQYHHFRHRLPLARVRLVEVGGDVQLDSVRIFFSFSSDLQSTQASSLELTEISRENVPKSGTPQLACGLVASNLNLKPGECLRVRGEVAPDAKSFVLNLGKDSNNLCLHFNPRFNAHGDANTIVCNSKDGGAWGTEQREAVFPFQPGSVAEVCITFDQANLTVKLPDGYEFKFPNRLNLEAINYMAADGDFKIKCVAFD
Fig. S4 Mass spectrum of intact Gal-7–8S–Gal-1 (calculated: 33077.24 Da, experimental: 33185.2 Da) (a), peptide mass fingerprint (b) and extent of sequence coverage (c). Detected peptides in the given sequences add up to a sequence coverage of 96.4%. The sequence stretch of the inserted 33-amino-acid linker of Gal-8S is highlighted in green.

## Slide 5
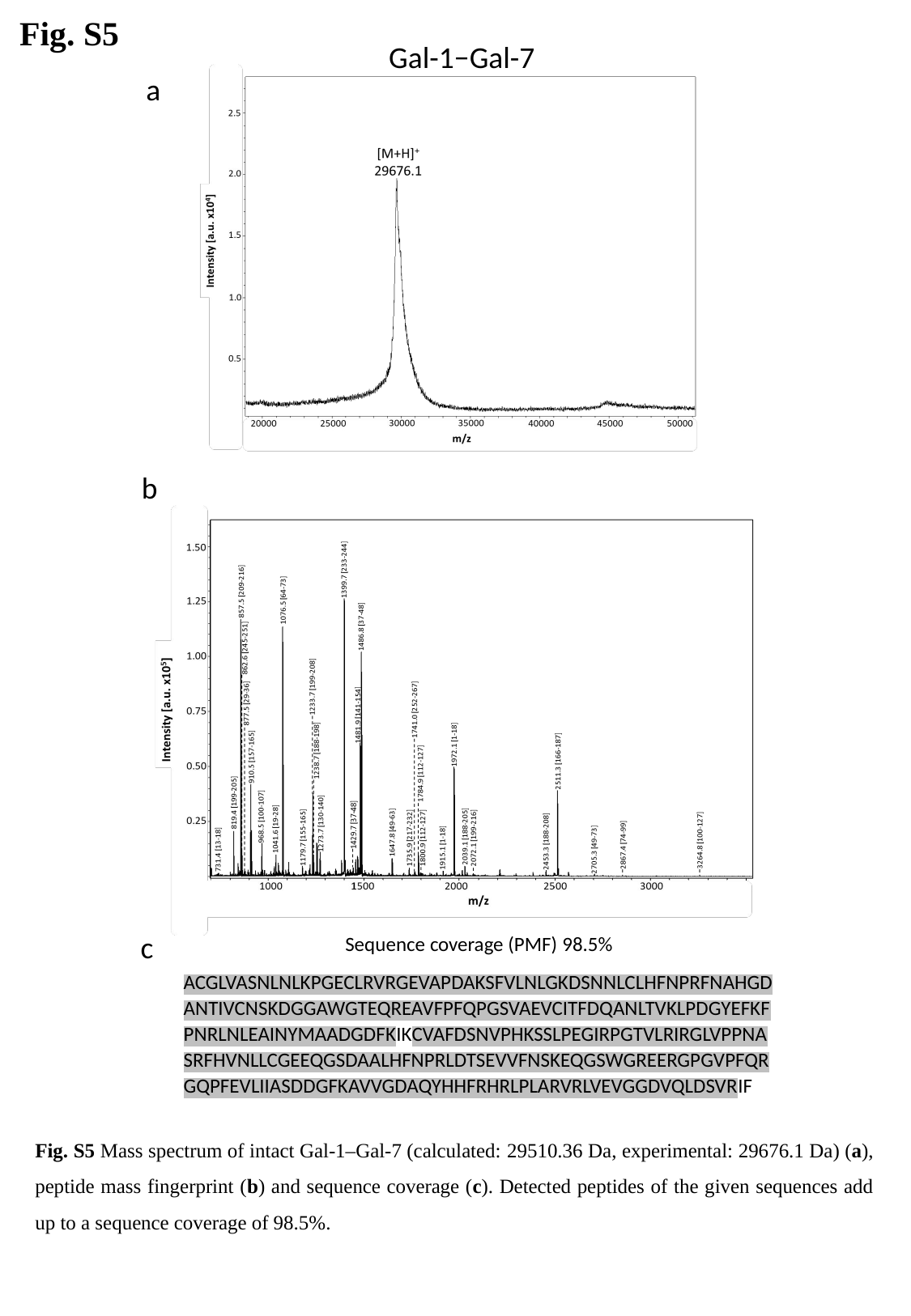

Fig. S5
Gal-1−Gal-7
a
b
c
Sequence coverage (PMF) 98.5%
ACGLVASNLNLKPGECLRVRGEVAPDAKSFVLNLGKDSNNLCLHFNPRFNAHGDANTIVCNSKDGGAWGTEQREAVFPFQPGSVAEVCITFDQANLTVKLPDGYEFKFPNRLNLEAINYMAADGDFKIKCVAFDSNVPHKSSLPEGIRPGTVLRIRGLVPPNASRFHVNLLCGEEQGSDAALHFNPRLDTSEVVFNSKEQGSWGREERGPGVPFQRGQPFEVLIIASDDGFKAVVGDAQYHHFRHRLPLARVRLVEVGGDVQLDSVRIF
Fig. S5 Mass spectrum of intact Gal-1–Gal-7 (calculated: 29510.36 Da, experimental: 29676.1 Da) (a), peptide mass fingerprint (b) and sequence coverage (c). Detected peptides of the given sequences add up to a sequence coverage of 98.5%.

## Slide 6
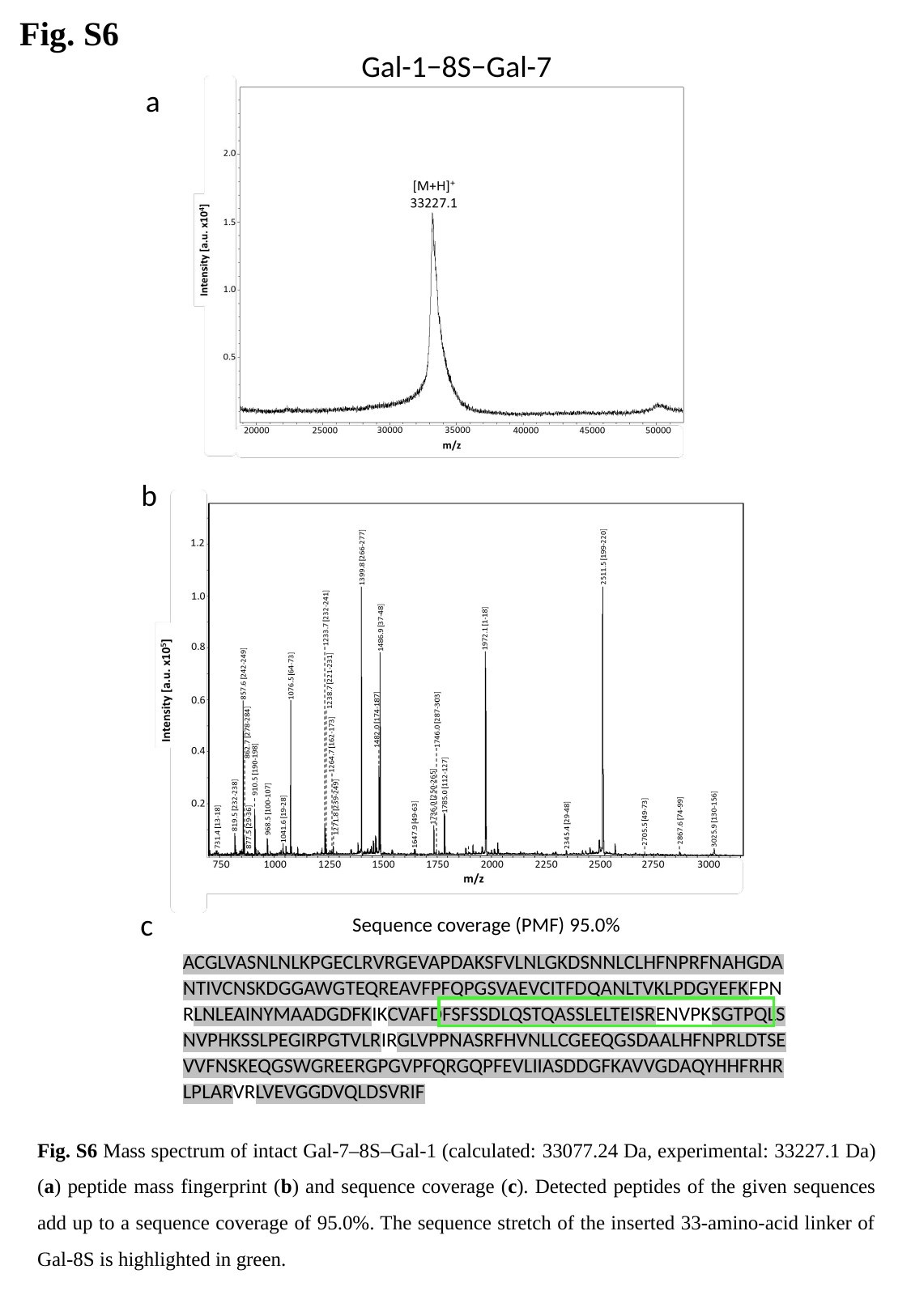

Fig. S6
Gal-1−8S−Gal-7
a
b
c
Sequence coverage (PMF) 95.0%
ACGLVASNLNLKPGECLRVRGEVAPDAKSFVLNLGKDSNNLCLHFNPRFNAHGDANTIVCNSKDGGAWGTEQREAVFPFQPGSVAEVCITFDQANLTVKLPDGYEFKFPNRLNLEAINYMAADGDFKIKCVAFDFSFSSDLQSTQASSLELTEISRENVPKSGTPQLSNVPHKSSLPEGIRPGTVLRIRGLVPPNASRFHVNLLCGEEQGSDAALHFNPRLDTSEVVFNSKEQGSWGREERGPGVPFQRGQPFEVLIIASDDGFKAVVGDAQYHHFRHRLPLARVRLVEVGGDVQLDSVRIF
Fig. S6 Mass spectrum of intact Gal-7–8S–Gal-1 (calculated: 33077.24 Da, experimental: 33227.1 Da) (a) peptide mass fingerprint (b) and sequence coverage (c). Detected peptides of the given sequences add up to a sequence coverage of 95.0%. The sequence stretch of the inserted 33-amino-acid linker of Gal-8S is highlighted in green.

## Slide 7
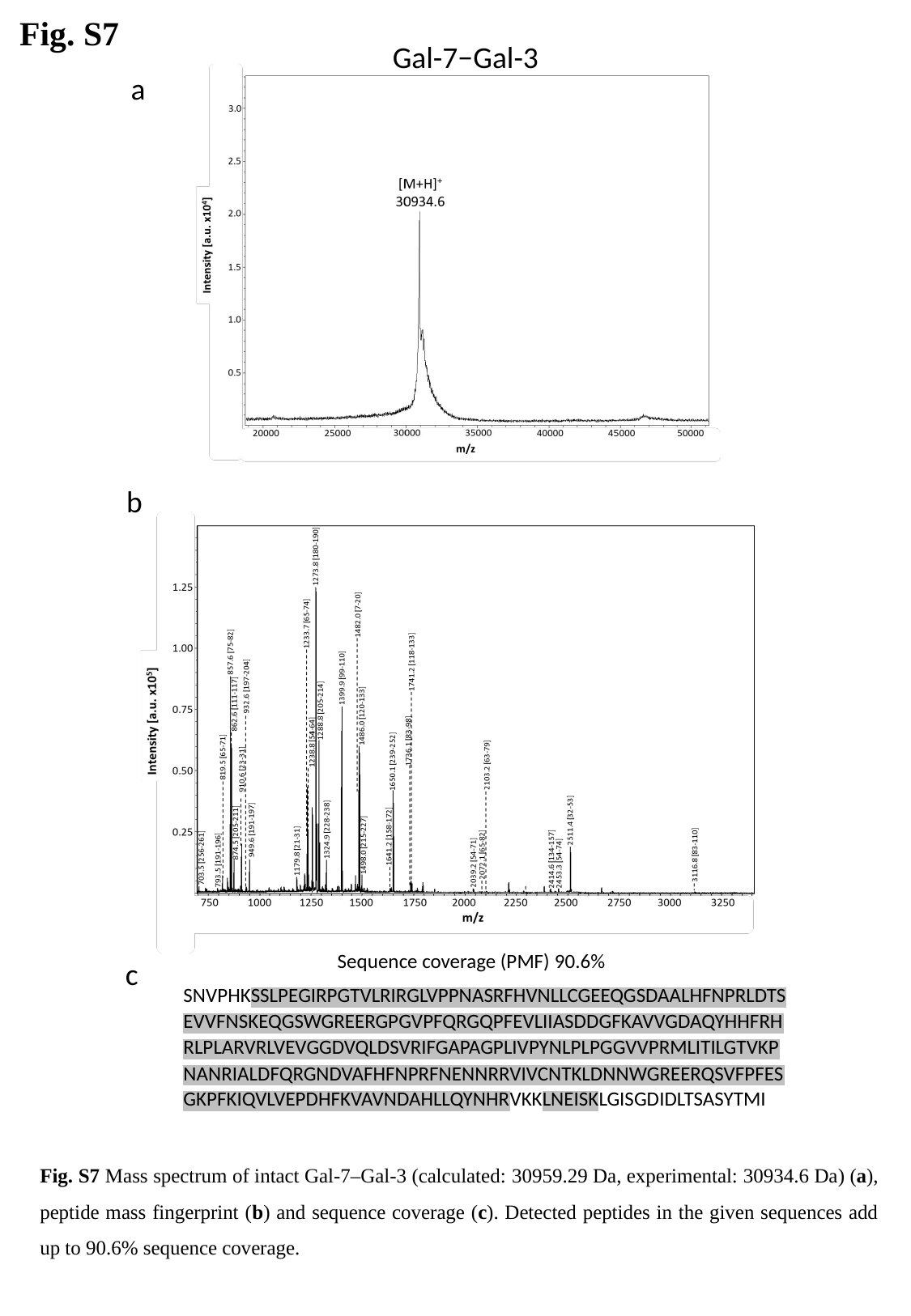

Fig. S7
Gal-7−Gal-3
a
b
Sequence coverage (PMF) 90.6%
c
SNVPHKSSLPEGIRPGTVLRIRGLVPPNASRFHVNLLCGEEQGSDAALHFNPRLDTSEVVFNSKEQGSWGREERGPGVPFQRGQPFEVLIIASDDGFKAVVGDAQYHHFRHRLPLARVRLVEVGGDVQLDSVRIFGAPAGPLIVPYNLPLPGGVVPRMLITILGTVKPNANRIALDFQRGNDVAFHFNPRFNENNRRVIVCNTKLDNNWGREERQSVFPFESGKPFKIQVLVEPDHFKVAVNDAHLLQYNHRVKKLNEISKLGISGDIDLTSASYTMI
Fig. S7 Mass spectrum of intact Gal-7–Gal-3 (calculated: 30959.29 Da, experimental: 30934.6 Da) (a), peptide mass fingerprint (b) and sequence coverage (c). Detected peptides in the given sequences add up to 90.6% sequence coverage.

## Slide 8
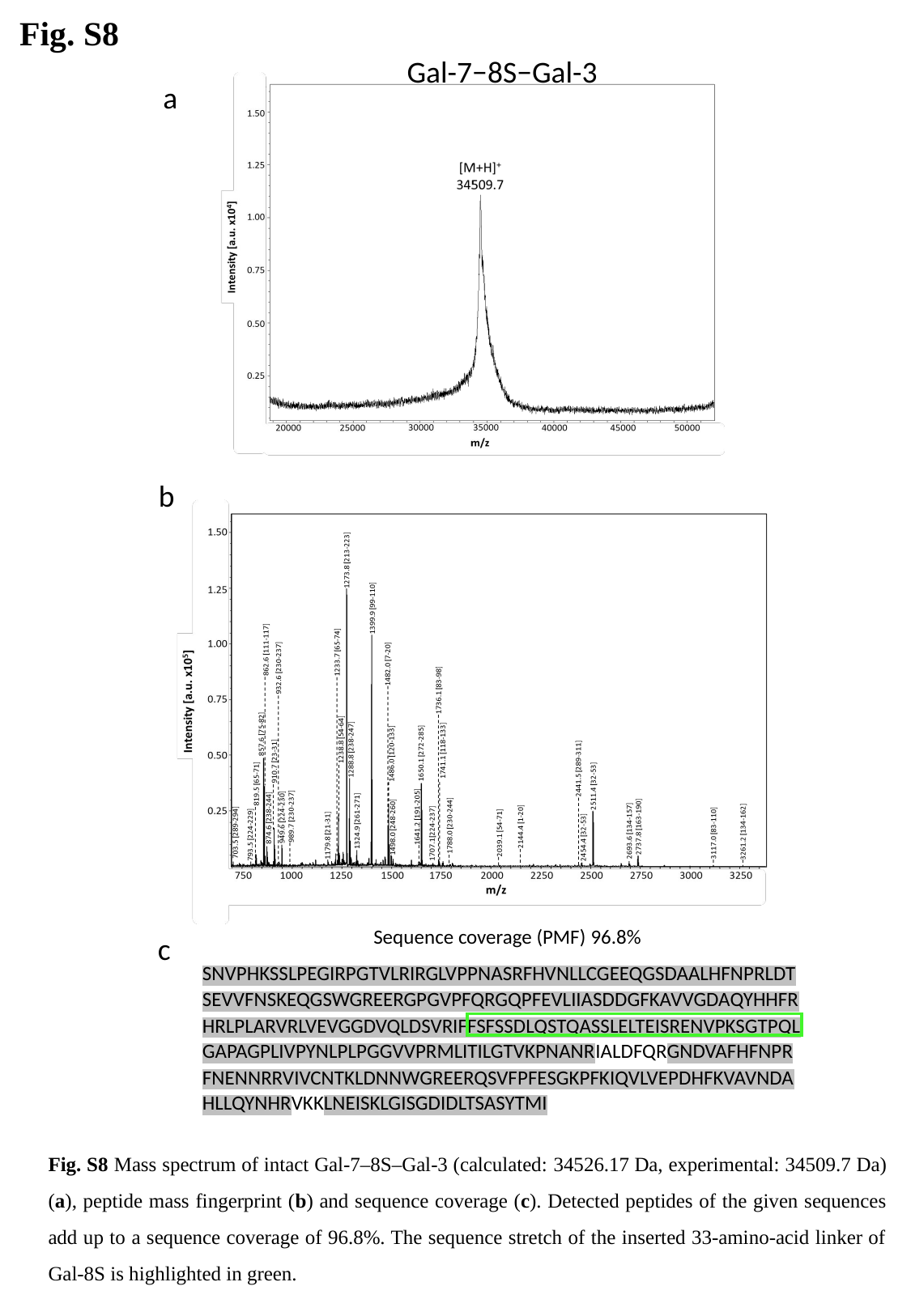

Fig. S8
Gal-7−8S−Gal-3
a
b
Sequence coverage (PMF) 96.8%
c
SNVPHKSSLPEGIRPGTVLRIRGLVPPNASRFHVNLLCGEEQGSDAALHFNPRLDTSEVVFNSKEQGSWGREERGPGVPFQRGQPFEVLIIASDDGFKAVVGDAQYHHFRHRLPLARVRLVEVGGDVQLDSVRIFFSFSSDLQSTQASSLELTEISRENVPKSGTPQLGAPAGPLIVPYNLPLPGGVVPRMLITILGTVKPNANRIALDFQRGNDVAFHFNPRFNENNRRVIVCNTKLDNNWGREERQSVFPFESGKPFKIQVLVEPDHFKVAVNDAHLLQYNHRVKKLNEISKLGISGDIDLTSASYTMI
Fig. S8 Mass spectrum of intact Gal-7–8S–Gal-3 (calculated: 34526.17 Da, experimental: 34509.7 Da) (a), peptide mass fingerprint (b) and sequence coverage (c). Detected peptides of the given sequences add up to a sequence coverage of 96.8%. The sequence stretch of the inserted 33-amino-acid linker of Gal-8S is highlighted in green.

## Slide 9
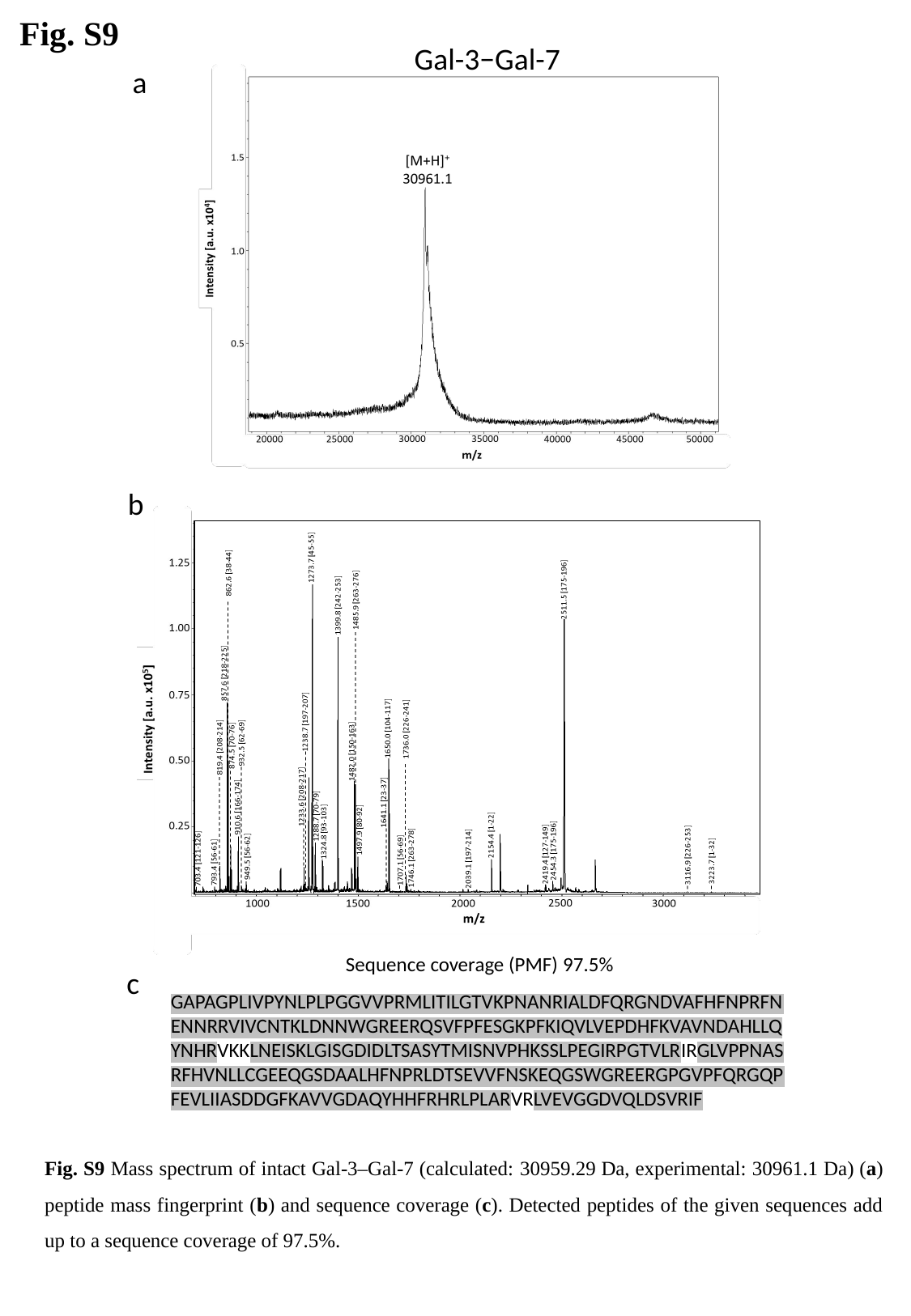

Fig. S9
Gal-3−Gal-7
a
b
Sequence coverage (PMF) 97.5%
c
GAPAGPLIVPYNLPLPGGVVPRMLITILGTVKPNANRIALDFQRGNDVAFHFNPRFNENNRRVIVCNTKLDNNWGREERQSVFPFESGKPFKIQVLVEPDHFKVAVNDAHLLQYNHRVKKLNEISKLGISGDIDLTSASYTMISNVPHKSSLPEGIRPGTVLRIRGLVPPNASRFHVNLLCGEEQGSDAALHFNPRLDTSEVVFNSKEQGSWGREERGPGVPFQRGQPFEVLIIASDDGFKAVVGDAQYHHFRHRLPLARVRLVEVGGDVQLDSVRIF
Fig. S9 Mass spectrum of intact Gal-3–Gal-7 (calculated: 30959.29 Da, experimental: 30961.1 Da) (a) peptide mass fingerprint (b) and sequence coverage (c). Detected peptides of the given sequences add up to a sequence coverage of 97.5%.

## Slide 10
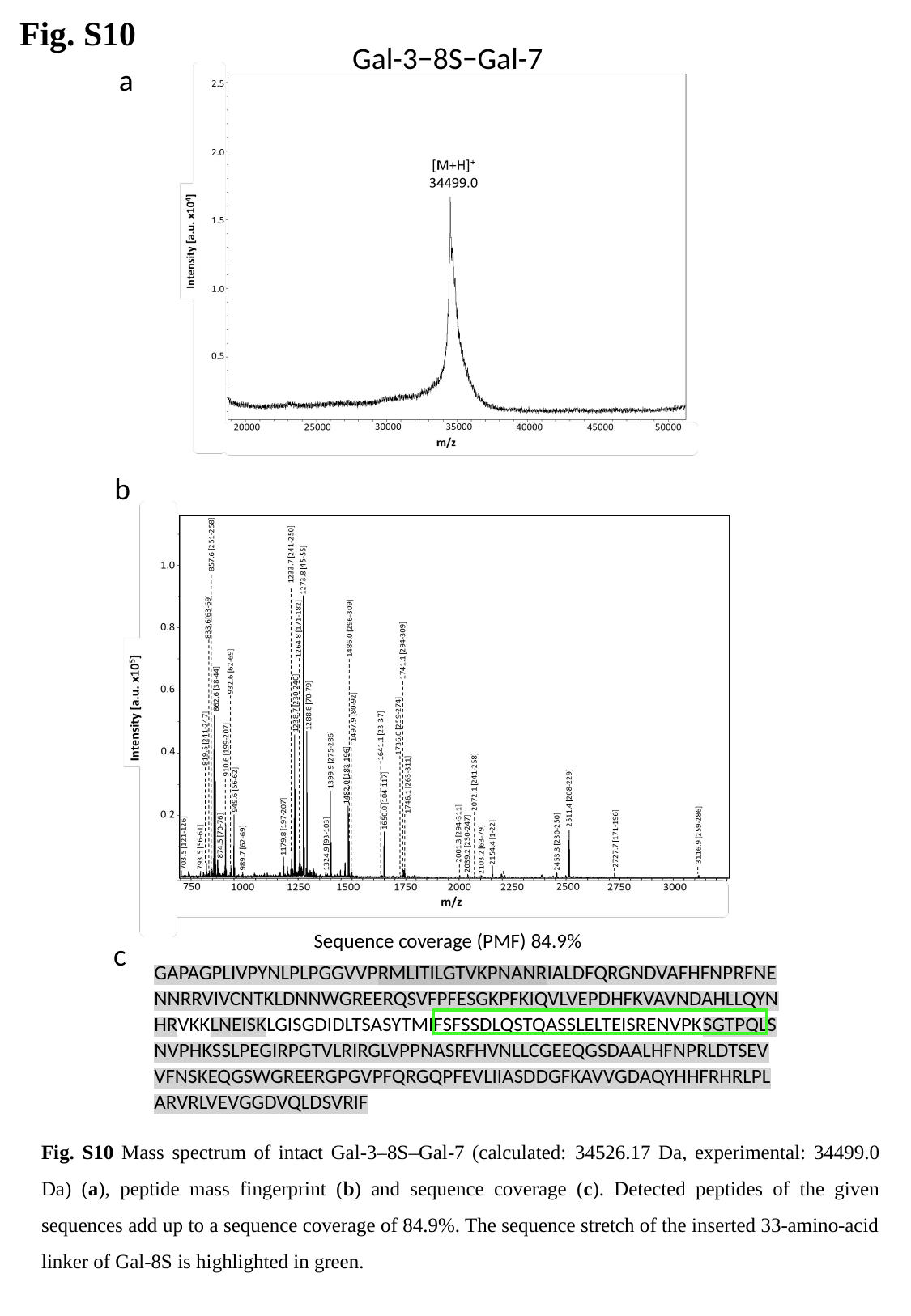

Fig. S10
Gal-3−8S−Gal-7
a
b
Sequence coverage (PMF) 84.9%
c
GAPAGPLIVPYNLPLPGGVVPRMLITILGTVKPNANRIALDFQRGNDVAFHFNPRFNENNRRVIVCNTKLDNNWGREERQSVFPFESGKPFKIQVLVEPDHFKVAVNDAHLLQYNHRVKKLNEISKLGISGDIDLTSASYTMIFSFSSDLQSTQASSLELTEISRENVPKSGTPQLSNVPHKSSLPEGIRPGTVLRIRGLVPPNASRFHVNLLCGEEQGSDAALHFNPRLDTSEVVFNSKEQGSWGREERGPGVPFQRGQPFEVLIIASDDGFKAVVGDAQYHHFRHRLPLARVRLVEVGGDVQLDSVRIF
Fig. S10 Mass spectrum of intact Gal-3–8S–Gal-7 (calculated: 34526.17 Da, experimental: 34499.0 Da) (a), peptide mass fingerprint (b) and sequence coverage (c). Detected peptides of the given sequences add up to a sequence coverage of 84.9%. The sequence stretch of the inserted 33-amino-acid linker of Gal-8S is highlighted in green.

## Slide 11
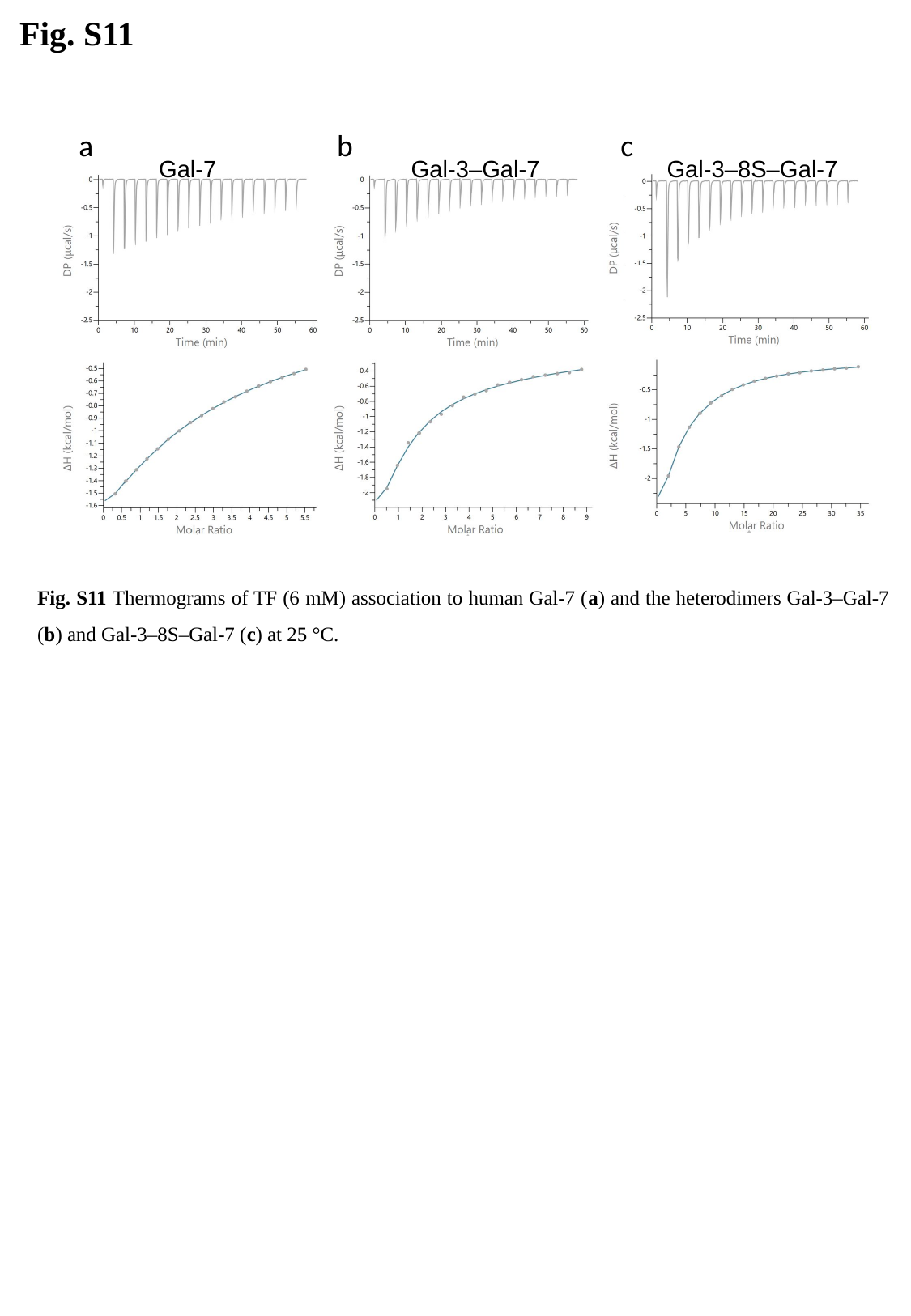

Fig. S11
a
b
c
Gal-7
Gal-3–Gal-7
Gal-3–8S–Gal-7
Fig. S11 Thermograms of TF (6 mM) association to human Gal-7 (a) and the heterodimers Gal-3–Gal-7 (b) and Gal-3–8S–Gal-7 (c) at 25 °C.

## Slide 12
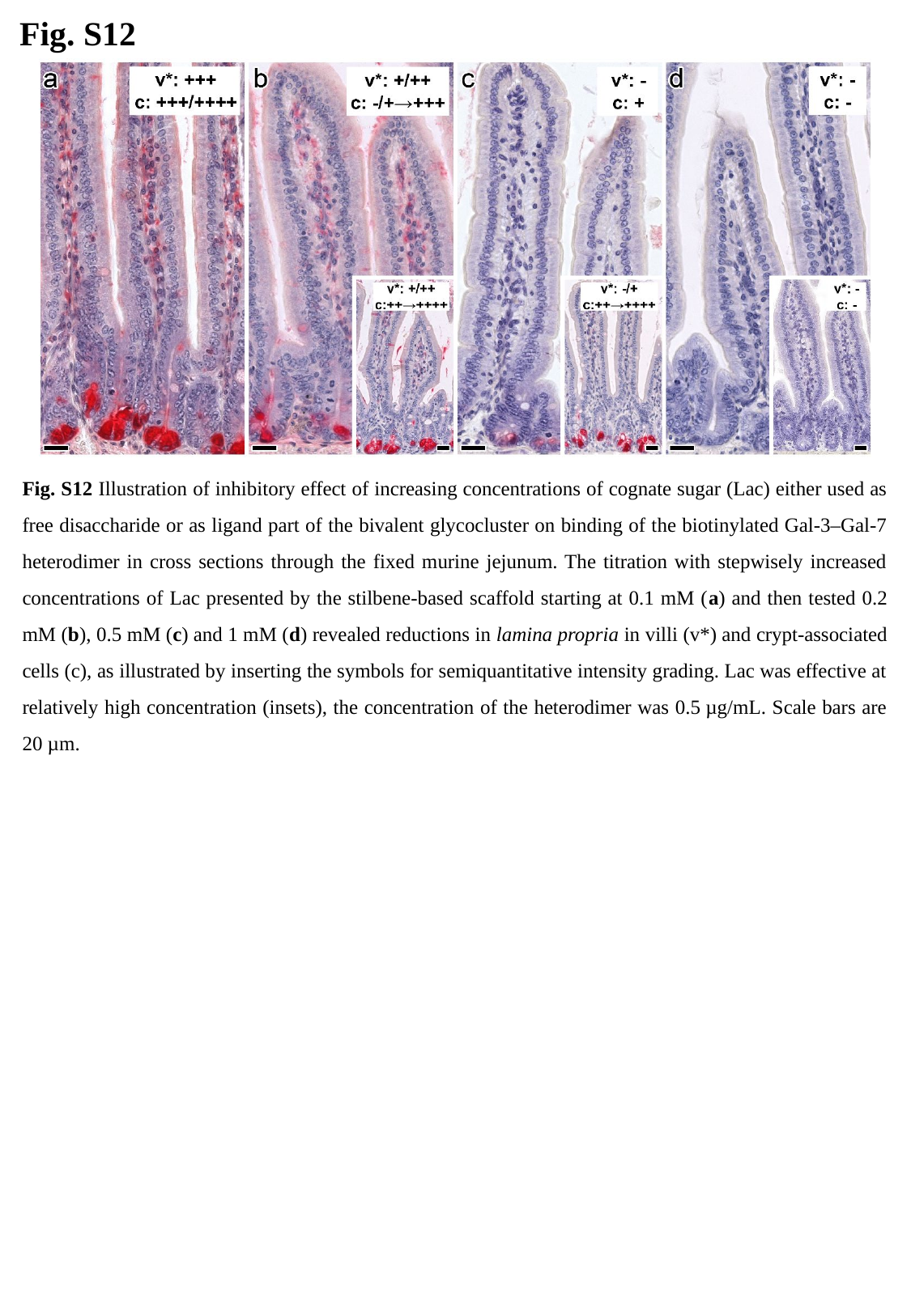

Fig. S12
Fig. S12 Illustration of inhibitory effect of increasing concentrations of cognate sugar (Lac) either used as free disaccharide or as ligand part of the bivalent glycocluster on binding of the biotinylated Gal-3–Gal-7 heterodimer in cross sections through the fixed murine jejunum. The titration with stepwisely increased concentrations of Lac presented by the stilbene-based scaffold starting at 0.1 mM (a) and then tested 0.2 mM (b), 0.5 mM (c) and 1 mM (d) revealed reductions in lamina propria in villi (v*) and crypt-associated cells (c), as illustrated by inserting the symbols for semiquantitative intensity grading. Lac was effective at relatively high concentration (insets), the concentration of the heterodimer was 0.5 µg/mL. Scale bars are 20 µm.

## Slide 13
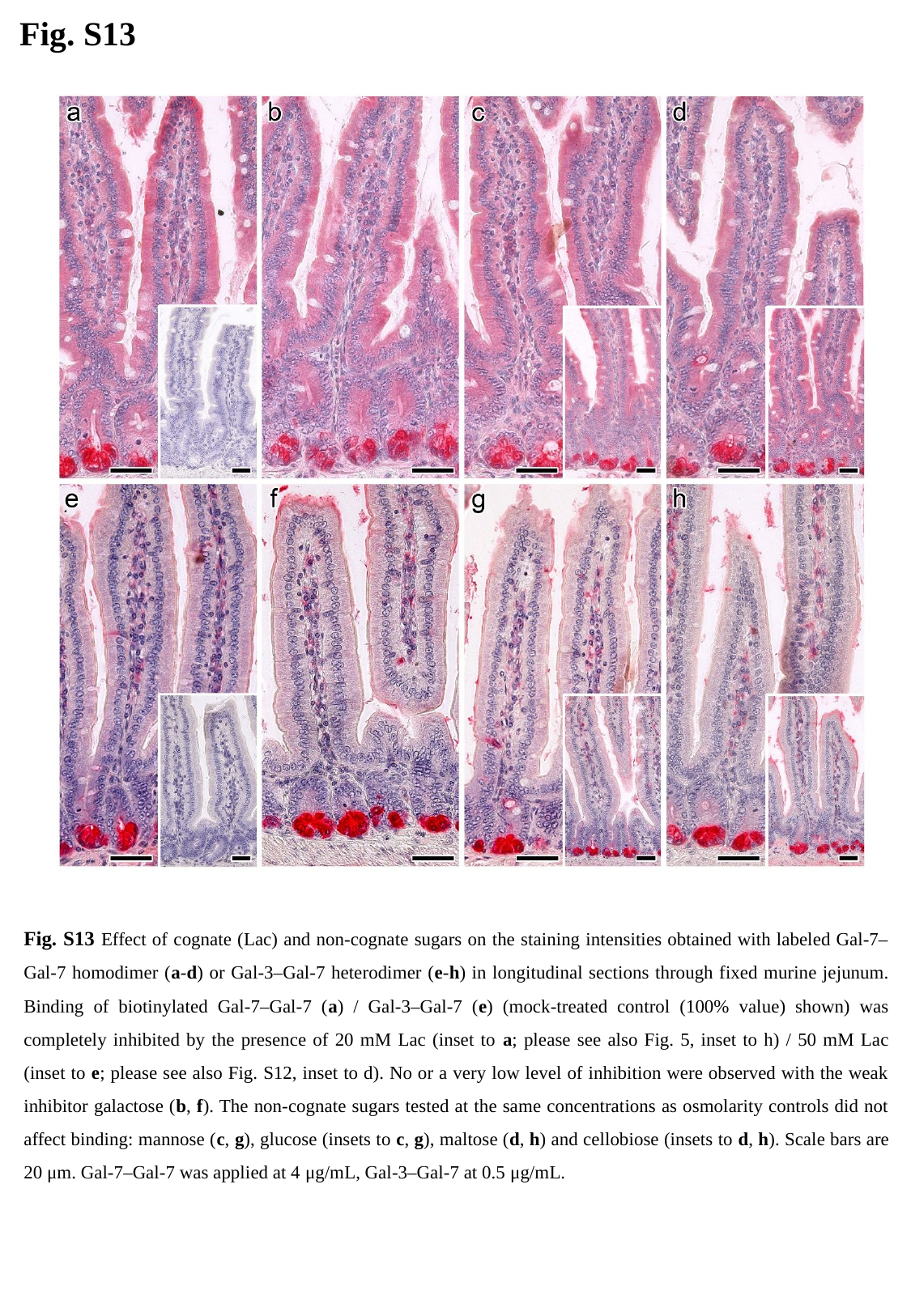

Fig. S13
Fig. S13 Effect of cognate (Lac) and non-cognate sugars on the staining intensities obtained with labeled Gal-7–Gal-7 homodimer (a-d) or Gal-3–Gal-7 heterodimer (e-h) in longitudinal sections through fixed murine jejunum. Binding of biotinylated Gal-7–Gal-7 (a) / Gal-3–Gal-7 (e) (mock-treated control (100% value) shown) was completely inhibited by the presence of 20 mM Lac (inset to a; please see also Fig. 5, inset to h) / 50 mM Lac (inset to e; please see also Fig. S12, inset to d). No or a very low level of inhibition were observed with the weak inhibitor galactose (b, f). The non-cognate sugars tested at the same concentrations as osmolarity controls did not affect binding: mannose (c, g), glucose (insets to c, g), maltose (d, h) and cellobiose (insets to d, h). Scale bars are 20 μm. Gal-7–Gal-7 was applied at 4 μg/mL, Gal-3–Gal-7 at 0.5 μg/mL.

## Slide 14
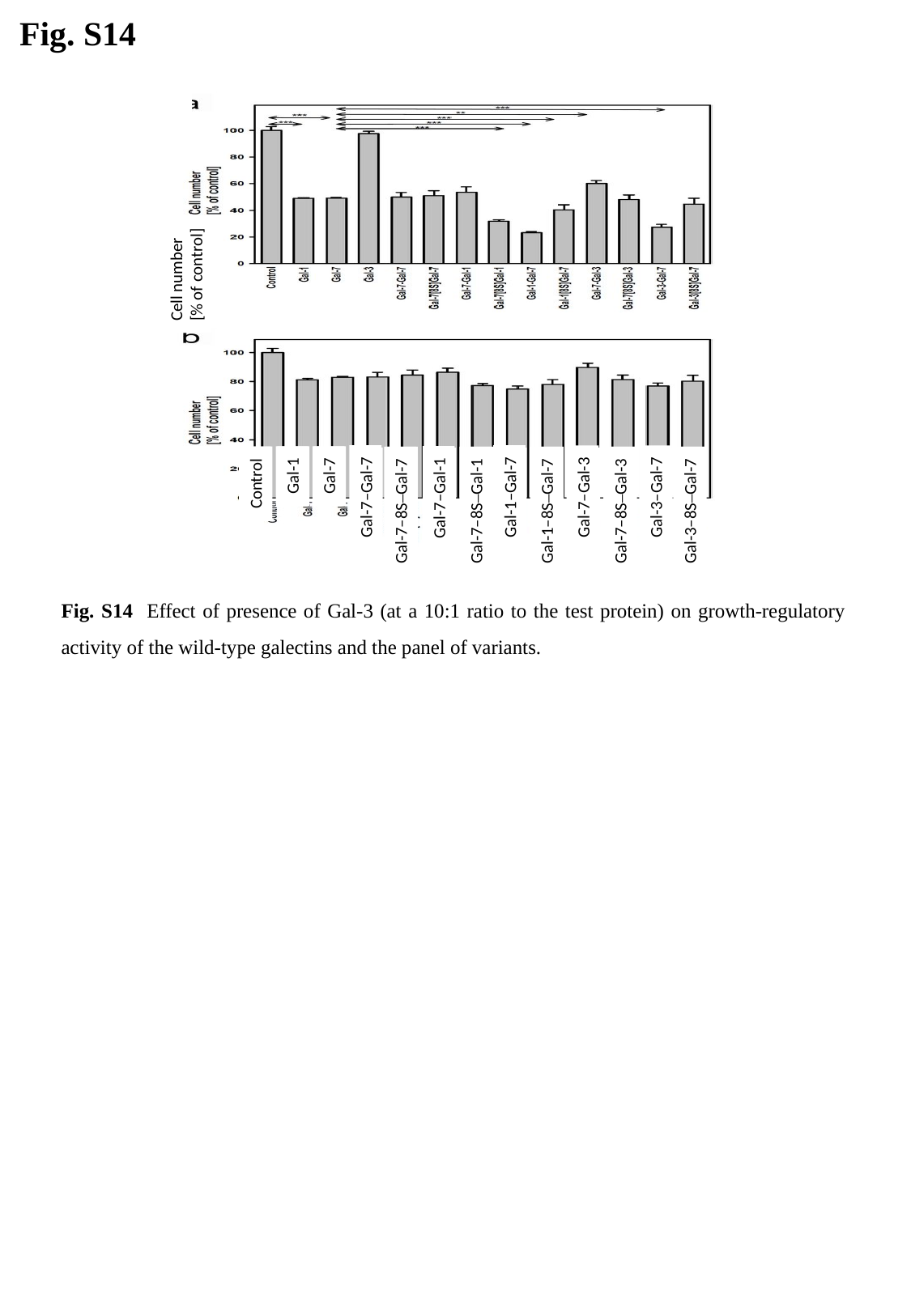

Fig. S14
Cell number
[% of control]
Gal-1
Gal-1
Gal-7
Gal-7
Control
Control
Gal-7–Gal-7
Gal-1–Gal-7
Gal-7–Gal-3
Gal-3–Gal-7
Gal-7–Gal-1
Gal-7–Gal-3
Gal-7–Gal-7
Gal-7–Gal-1
Gal-1–Gal-7
Gal-3–Gal-7
Gal-7–8S–Gal-7
Gal-7–8S–Gal-7
Gal-7–8S–Gal-1
Gal-7–8S–Gal-1
Gal-1–8S–Gal-7
Gal-1–8S–Gal-7
Gal-7–8S–Gal-3
Gal-7–8S–Gal-3
Gal-3–8S–Gal-7
Gal-3–8S–Gal-7
Fig. S14 Effect of presence of Gal-3 (at a 10:1 ratio to the test protein) on growth-regulatory activity of the wild-type galectins and the panel of variants.
